# OCT4 maintains self-renewal and reverses senescence in human hair follicle mesenchymal stem cells through the downregulation of p21 by DNA methyltransferases

**DOI:** 10.1186/s13287-018-1120-x

**Published:** 2019-01-15

**Authors:** Yan Lu, Huinan Qu, Da Qi, Wenhong Xu, Shutong Liu, Xiangshu Jin, Peiye Song, Yantong Guo, Yiyang Jia, Xinqi Wang, Hairi Li, Yulin Li, Chengshi Quan

**Affiliations:** 1 The Key Laboratory of Pathobiology, Ministry of Education, Department of Pathology, College of Basic Medical Sciences, Jilin University, 126 Xinmin Avenue, Changchun, 130021 China; 2 Cell Processing Section, Department of Transfusion, Clinical Center, National Institutes of Health, Bethesda, MD 20892 USA; 3 Department of Cellular and Molecular Medicine, University of California, San Diego, CA 92093-0651 USA

**Keywords:** hHFMSCs, OCT4, Self-renewal, Senescence, p21, DNMTs

## Abstract

**Background:**

Self-renewal is dependent on an intrinsic gene regulatory network centered on OCT4 and on an atypical cell cycle G1/S transition, which is also regulated by OCT4. p21, a gene negatively associated with self-renewal and a senescence marker, is a member of the universal cyclin-dependent kinase inhibitors (CDKIs) and plays critical roles in the regulation of the G1/S transition. The expression of p21 can be regulated by OCT4-targeted DNA methyltransferases (DNMTs), which play distinct roles in gene regulation and maintaining pluripotency properties. The aim of this study was to determine the role of OCT4 in the regulation of self-renewal and senescence in human hair follicle mesenchymal stem cells (hHFMSCs) and to characterize the molecular mechanisms involved.

**Methods:**

A lentiviral vector was used to ectopically express OCT4. The influences of OCT4 on the self-renewal and senescence of hHFMSCs were investigated. Next-generation sequencing (NGS) was performed to identify the downstream genes of OCT4 in this process. Methylation-specific PCR (MSP) analysis was performed to measure the methylation level of the p21 promoter region. p21 was overexpressed in hHFMSCs^OCT4^ to test its downstream effect on OCT4. The regulatory effect of OCT4 on DNMTs was examined by ChIP assay. 5-aza-dC/zebularine was used to inhibit the expression of DNMTs, and then self-renewal properties and senescence in hHFMSCs were detected.

**Results:**

The overexpression of OCT4 promoted proliferation, cell cycle progression, and osteogenic differentiation capacity of hHFMSCs. The cell senescence of hHFMSCs was markedly suppressed due to the ectopic expression of OCT4. Through NGS, we identified 2466 differentially expressed genes (DEGs) between hHFMSCs^OCT4^ and hHFMSCs^EGFP^, including p21, which was downregulated. The overexpression of p21 abrogated the proliferation and osteogenic differentiation capacity of hHFMSCs^OCT4^ and promoted cell senescence. OCT4 enhanced the transcription of DNMT genes, leading to an elevation in the methylation of the p21 promoter. The inhibition of DNMTs reversed the OCT4-induced p21 reduction, depleted the self-renewal of hHFMSCs^OCT4^, and triggered cell senescence.

**Conclusions:**

OCT4 maintains the self-renewal ability of hHFMSCs and reverses senescence by suppressing the expression of p21 through the upregulation of DNMTs.

**Electronic supplementary material:**

The online version of this article (10.1186/s13287-018-1120-x) contains supplementary material, which is available to authorized users.

## Background

As a regenerative organ, hair follicles are characterized by periodic growth and constant self-renewal [1]. Dermal sheath cells and dermal papilla cells in the hair follicles share a similar stemness to bone marrow mesenchymal stem cells [2]. Human hair follicle mesenchymal stem cells (hHFMSCs) express relatively specific surface markers of mesenchymal stem cells (MSCs) and have the potential to differentiate into osteoblasts, adipocytes, and chondrocytes [3]. Due to the abundance of supplies, safe access, and low immunogenicity [4], hHFMSCs are considered to be a promising source of cells for the rapidly emerging field of regenerative medicine and to be particularly beneficial for personalized cell therapy. However, hHFMSCs alter their biological properties and enter into a state of replicative senescence after a certain period of cell culture. Thus, fully understanding the molecular mechanisms involved in the regulation of hHFMSC self-renewal and senescence (i.e., maintaining stem cell properties) would provide a great advancement in the application of these cells. The regulation of stem cell self-renewal and its properties resides in conserved transcriptional regulatory networks [5, 6] and epigenetic modifications, such as DNA methylation, that work together to repress developmental genes and activate stemness genes [7].

OCT4 (also known as POU domain transcription factor, Pou5f1) is the most upstream gene in the signaling pathway that regulates the self-renewal of stem cells [8]. OCT4, downregulated after cell differentiation [9], maintains cell self-renewal in the absence of feeder cells in human and mouse embryonic stem cells (ESCs) [10]. The abrogation of OCT4 in ESCs leads to cell differentiation [11–13]. OCT4, SOX2, and NANOG are well-known key components of the core regulatory network that governs stem cells/pluripotency, whereas epigenetic regulators also play important roles in maintaining pluripotency [14, 15]. Moreover, NANOG expression is directly regulated by OCT4 and SOX2 [16]. The phenotypes of SOX2-null cells can be rescued by ectopic expression of wild-type levels of OCT4 [17]. Therefore, OCT4 is considered to be the paramount transcription factor (TF) in the establishment and maintenance of pluripotency. There is a strong inverse correlation between the expression of OCT4 and senescence [18]. OCT4 is also a cell cycle promoter, which removes the blockage of cell cycle progression in the G1 phase and stimulates the entry into the S phase [19]; this is a unique cell cycle structure that is required by self-renewal capacity [20, 21].

It has been well characterized that the G1/S transition is mainly regulated by G1 regulatory molecules [22]. Among these regulatory molecules, p21 is a member of the universal cyclin-dependent kinase inhibitors (CDKIs) and plays critical roles in the regulation of the G1/S transition [23]. p21 mediates cell quiescence in neural [24] and hair follicle stem cells [25]. The knockdown of p21 enhances the proliferation, expression of stemness markers, and osteogenic potential in human MSCs [26]. It was reported that the gene transcription level of p21 was regulated by DNA methylation because the p21 promoter contained a high density of potentially methylatable CpG dinucleotide clusters [27].

DNA methylation is a heritable chemical modification of the DNA molecule, is predominantly associated with transcriptional repression, and is essential for mammalian development [28]. DNA methyltransferases (DNMTs) lead to the ectopic methylation of DNAs, leading to gene silencing. DNA methyltransferase 3 alpha (DNMT3a) and beta (DNMT3b) establish de novo methylation in early development [29]. Once established, methylation patterns are faithfully maintained by DNA methyltransferase 1 (DNMT1) following mitosis in cells [30]. It was reported that DNMT3a^−/−^ and DNMT3b^−/−^ double-knockout ESCs are fully viable and could be differentiated into all three germ layers [31, 32]. Interestingly, rapid downregulation of exogenous DNMT1 results in subsequent cell death. Thus, it seems that DNMT1 is essential to maintain the viability of hESCs [33]. DNA methylation plays multiple roles in developmental gene regulation in mammalian ESCs [34].

The present study investigated the role of OCT4 in maintaining the self-renewal ability of hHFMSCs and reversing senescence, and the mechanism involved p21 and DNMTs. These results support the application of hHFMSCs in regenerative medicine, particularly to benefit personalized cell therapy.

## Materials and methods

### Cell culture and reagents

hHFMSCs were isolated and identified as reported in our previous work [35]. hHFMSCs and transduced hHFMSCs (hHFMSCs^EGFP^ and hHFMSCs^OCT4^) were cultured in H-DMEM/F12 (Gibco, Grand Island, NY, USA) medium supplemented with 10% FBS (Gibco), 10 ng/ml bFGF (R&D Systems, Minneapolis, MN, USA), and 100 U/ml penicillin-streptomycin (Hyclone, Logan, UT, USA). HEK293T cells were cultured in H-DMEM (Gibco) supplemented with 10% FBS at 37 °C and in the presence of 5% CO_2_. The following antibodies were used: anti-OCT4, anti-DNMT1, and anti-Ki67 antibodies (Abcam, Cambridge, MA, USA); anti-DNMT3a, anti-p21, anti-proliferating cell nuclear antigen (PCNA), and anti-Cyclin D1 antibodies (Cell Signaling Technology, Beverly, MA, USA); and anti-DNMT3b antibody (Thermo Fisher Scientific, Waltham, MA, USA). 5-aza-dC and zebularine were purchased from Sigma-Aldrich (St. Louis, MO, USA).

### Lentivirus production and lentivirus transduction

The lentiviral vectors pLV-EF1α-IRES-EGFP and pLV-EF1α-OCT4-IRES-EGFP and the packaging plasmids expressing gag-pol, pVSVG, and rev genes were obtained from the Institute of Biochemistry and Cell Biology of Shanghai Life Science Research Institute, Chinese Academy of Sciences. These vectors were transfected into 293T cells by X-tremeGene HP DNA Transfection Reagent (Roche Applied Science, Rotkreuz, CH). Viral supernatants were harvested at 48 h and 72 h after transfection and concentrated by ultracentrifugation. hHFMSCs-P15 (1 × 10^4^/well) were seeded on Matrigel-coated 12-well plates, and cells were infected with lentivirus in the presence of 8 μg/ml polybrene (Santa Cruz, CA, USA) for 24 h.

After transduction, live cells were identified by 7-amino actinomycin (7AAD, KeyGEN BioTECH, Nanjing, China) exclusion and analyzed for GFP expression on a BD FACSCalibur flow cytometer (BD Biosciences, San Jose, CA, USA) to detect transduction efficiency.

### Real-time fluorescence quantitative polymerase chain reaction (RT-qPCR)

Total RNA was isolated by TRIzol Reagent (Invitrogen Life Technologies, NY, USA). RNA was then subjected to cDNA synthesis using M/MLV kit (TAKARA, Osaka, Japan). RT-qPCR was performed with an ABI 7300 real-time PCR system (Applied Biosystems, Foster City, CA, USA), and samples were normalized to GAPDH with the autoset baseline. qPCR was conducted in triplicates using 25 ng of reverse-transcribed cDNA and 0.2 μM of primer in a 20μl final reaction volume containing 1 × SYBR Premix EX Taq (TAKARA). Using comparative critical cycle (Ct) method with GAPDH as an endogenous control, the relative expression was calculated as 2 ^−ΔCt^ and compared between two groups. Detailed information on these primers is listed in Table 1.

**Table 1:** Primers for qPCR, ChIP, and MSP

Gene	Forward primers (5′ to 3′)	Reverse primers (5′ to 3′)
OCT4	CTGAAGCAGAAGAGGATCAC	GACCACATCCTTCTCGAGCC
p21	TGGAGACTCTCAGGGTCGAAA	GGCGTTTGGAGTAGAAATC
p16	GTGGACCTGGCTGAGGAG	CTTTCAATCGGGGATGTCTG
hTERT	CATGGGCACGTCCGCAA	GGCGTGGTGGCACATGAA
DNMT1	ACCGCTTCTACTTCCTCGAGGCCTA	GTTGCAGTCCTCTGTGAACACTGTGG
DNMT3a	CACACAGAAGCATATCCAGGAGTG	AGTGGACTGGGAAACCAAATACCC
DNMT3b	AATGTGAATCCAGCCAGGAAAGGC	ACTGGATTACACTCCAGGAACCGT
GAPDH	CCATGTTCGTCATGGGTGTGA	CATGGACTGTGGTCATGAGT
DNMT1 (ChIP)	CCCCACACACTGGGTATAGAA	CGAGGCATTCATTCATTCATT
DNMT3a (ChIP)	TCATTTCATTCAACCTCCCAC	CTCTGAGATGAGCTGCCTTGA
DNMT3b (ChIP)	CATCCAAAGAATTAACCCTTCACT	GGTCTCCAAGTGCTTATAGTGGAT
p21 U (MSP)	TTTTGGGATTGGTTGGTTTG	ACACCCAACTCCAACTCCAC
p21 M (MSP)	TTGTAGTACGCGAGGTTTCG	CAACTCAACGCGACCCTAAT

### Western blotting analysis

Cells were harvested and lysed in 300 μl ice-cold radioimmunoprecipitation assay lysis buffer supplemented with 1% phenylmethylsulfonyl fluoride and then centrifuged at 12,000*g* at 4 °C for 30 min to remove cell debris. Protein concentration was determined by using a BCA Protein Assay Kit (Pierce Chemical Co., Rockford, IL, USA). Cell lysates were separated on 10% SDS-PAGE and then transferred onto a nitrocellulose membrane (Millipore, Temecula, CA, USA). The membrane was incubated with 5% nonfat milk (Applichem) at 37 °C for 1 h and then incubated with primary antibodies at 4 °C overnight and secondary antibodies conjugated to horseradish peroxidase for 1 h under the room temperature. The membranes were finally stained with an ECL Western blotting system (GE, Fairfield, CT, USA).

### Immunofluorescence

Cells were fixed in 4% paraformaldehyde (Ding Guo, Beijing, China) for 10 min and permeabilized with 0.1% Triton X-100 (Sigma, St. Louis, USA) for 20 min under the room temperature. One percent BSA/PBS was used to block nonspecific binding. Cells were then incubated overnight with primary antibodies (OCT4 and Ki67, 1:500) at 4 °C. The secondary antibody was Alexa Fluor 647 anti-rabbit IgG (1:200 dilution; CST). The nuclei were counterstained with 5 μg/ml DAPI (Sigma-Aldrich) for 2 min in the dark, and cells were visualized with a laser scanning confocal microscope (Olympus, Tokyo, Japan).

### Flow cytometry

Cells were trypsinized and counted. Approximately 1 × 10^6^ cells were used for the test. Cells were rinsed with PBS by centrifugation at 4 °C, resuspended with 1% bovine serum albumin (BSA; Roche Diagnostics, Mannheim, Germany) in PBS, and incubated for 30 min. Next, the cells were incubated with primary antibody rabbit anti-OCT4 (1:100) for 30 min on ice, followed by incubation with the Alexa Fluor 647-conjugated secondary antibody for 30 min. The labeled cells were thoroughly washed with PBS and analyzed on a BD FACSCalibur flow cytometer. The primary antibody was omitted as a negative control.

### Cell proliferation analysis

#### iCELLigence cell proliferation analysis

Cells were seeded at a density of 1500 cells/well into E-Plate 8 (ACEA Biosciences, Inc., San Diego, CA) containing 450 μl medium per well and monitored for 7 days at 37 °C in a 5% CO_2_ atmosphere, with one change of fresh medium at day 4. Dynamic monitoring of the growth pattern was determined by the impedance-based iCELLigence system (Roche Applied Science, Germany). The cell index was derived from measured cell-electrode impedance that correlates with the number of cells and viability.

#### Population doubling time = *t* × {lg2/(lg*N*_*t*_ − lg*N*_0_)}

Cells were seeded in 24-well plates at a low density (5000 cells/well) and cultured as described above. The number of cells was counted every 24 h through the following 7 days. Three replicate counts were performed in each of three independent experiments (*t*, incubation time; *N*_*t*_, cell number after culturing for *t* days; *N*_0_, cell number of starting time).

#### Cell Counting Kit-8 (CCK8) assay

One hundred microliters cell suspensions (1000 cells/well) was dispensed in 96-well plates and monitored for 7 days at 37 °C in a 5% CO_2_ atmosphere, with one change of fresh medium at day 4. Following the treatment with 5-aza-dC at the concentrations of 100 μM for 72 h, or with 100 μM Zebularine for 48 h,10 μl CCK8 (Roche Diagnosis, Mannheim, Germany) was added to each well and incubated for 2 h. The absorbance at 450 nm (A450) was examined with a scanning multi-well spectrophotometer (TECAN, CH).

### Colony formation assay

hHFMSCs ^EGFP^ and hHFMSCs ^OCT4^ cells were seeded into 6-well plates in a cell density of 20 cells/cm^2^, and they were allowed to grow for 5 to 7 days until clones were visible. PBS-washed cells were fixed with 4% paraformaldehyde and then stained with Giemsa. The stained clones were counted, and the rate of clone’s formation was calculated as follows: (number of stained clones/number of seeded cells) × 100%.

### Sphere formation assay

Single cells were plated in ultra-low 6-well attachment plates (Corning, Lowell, MA, USA) at a density of 500 cells/cm^2^ in H-DMEM/F12 medium, supplemented with 1% penicillin/streptomycin, B27 (1:50, Invitrogen, Life Technologies Corp, Carlsbad, CA, USA), 5 μg/ml insulin (R&D Systems), 20 ng/ml epidermal growth factor (R&D Systems), and 20 ng/ml bFGF. After 5 days, sphere formation was assessed by counting the number of clonal spheres (cells > 3) under an optical microscope.

### Cell cycle assay

Cells were plated in 6-cm-diameter dishes (3 × 10^5^ cells/dish) and allowed to grow for 3 days. The cell cycle status was determined by the Cell Cycle Detection Kit (KeyGEN BioTECH). Briefly, cells were collected and washed with cooled PBS, fixed and permeabilized, treated with RNase A and stained with a PI solution, and then observed at 488 nm of excitation wavelength by flow cytometry and photographed.

$$ \mathrm{Proliferation}\ \mathrm{index}\ \left(\mathrm{PI}\right)=\left(\mathrm{S}+\mathrm{G}2\right)/\left(\mathrm{G}1+\mathrm{S}+\mathrm{G}2\right)\times 100\%. $$

### Osteogenic differentiation

Cells were cultured in the osteogenic medium (high glucose-DMEM, 10% FBS, 50 μM ascorbate-2-phosphate, 0.1 μM dexamethasone, 10 mM β-glycerolphosphate) for 2 weeks. Following the differentiation, Alizarin Red staining was performed to examine osteogenic differentiation.

### Transmission electron microscopy

5 × 10^7^ cells were rinsed with PBS and fixed for 30 min with 2.8% glutaraldehyde at room temperature. After rinsing twice, the specimens were post-fixed for 60 min with 2% osmium tetroxide in cacodylate buffer. The cells were then dehydrated in the increasing ethanol concentrations (40, 50, 70, 85, 90, 95, and 100%) and covered twice for 3 h with a thin layer of Araldite 502 resin (for ethanol substitution). Finally, the resin was allowed to polymerize at 60 °C for 48 h. The specimens were detached from the plastic vessels, inverted in embedding molds, covered with Araldite 502, and re-incubated at 60 °C for 48 h. The thin sections were prepared with an ultra-microtome (LKB-III, Sweden), contrasted with lead citrate and uranyl acetate, and observed in a blind fashion with a transmission electron microscope (Hitachi, JEM-1200EX, Japan). Each test was repeated three times. All reagents were purchased from Electron Microscopy Sciences (Cedarlane, Hornby, Ontario).

### Senescence-associated β-galactosidase (SA-β-gal) staining

Cells were seeded in 6-well plates with a density of 1 × 10^5^ cells/well. After cultured for 24 h, the cells were stained by SA-β-gal to detect cell senescence by using the SA-β-gal kit (Beyotime, Beijing, China) as described in the instruction of the manufacturer. The senescent cells were observed with an optical microscope and counted in five random fields of vision.

### Next-generation sequencing

The expression profile of mRNA (hHFMSCs^OCT4^ and hHFMSCs^EGFP^) was determined by next-generation sequencing (NGS). Total RNA from harvested cells was extracted by Trizol® Reagent (Invitrogen, USA) according to the manufacturer’s instructions. RNA quality and concentration were measured by using a NanoDrop spectrophotometer (Thermo Fisher Scientific, MA, USA) before further processing. Then RNA samples were shipped to the University of California (University of California, San Diego, CA, USA) for sequencing. The quality of extracted RNA was confirmed by using Agilent Bioanalyzer (Agilent Technology, USA) (RNA integrity number (RIN) > 8). After purification, the pooled libraries were loaded in eight lanes of flow cells and sequenced on a HiSeq 2500 (Illumina, Inc., San Diego, CA, USA) with V4 sequencing reagents. The reads were mapped to the human reference genome GRCh38/hg38 using Bowtie2. The expression values were calculated by using RSEM [36] for each gene (Ensembl, Homo.sapiens.GRCh38.87.gtf) in individual samples. Differential expression was analyzed between different subgroups using edgeR [37]. The criteria for differentially expressed mRNAs were set at a fold change of more than 2.0. Differential expression was analyzed between different subgroups using edgeR. Data were clustered and visualized using Cluster and Java TreeView.

### Gene ontology (GO) and Kyoto Encyclopedia of Genes and Genomes (KEGG) pathway mapping analysis

The Database for Annotation, Visualization and Integrated Discovery (DAVID) database [38] was used to analyze the differentially expressed genes between hHFMSCs^EGFP^ and hHFMSCs^OCT4^. The potential targets of differentially expressed genes were analyzed by the GO program, and enriched pathways were analyzed by the KEGG program. The GO analysis included biological process (BP), molecular function (MF), and cellular component (CC) terms, and *p* < 0.01 was regarded as statistically significant. The enriched pathways were analyzed by the KEGG program, and enriched pathways were identified according to *p* < 0.01 and FDR < 0.05.

### Chromatin immunoprecipitation (ChIP) assay

ChIP assays were performed with the EZ Magna ChIP G Chromatin Immunoprecipitation kit (Millipore, Billerica, MA, USA) using chromatin isolated from 1 × 10^6^ cells per condition, according to the manufacturer’s protocol. The anti-OCT4 (1:500) and anti-IgG (Millipore, as a negative control) antibodies were included. The specific primers for the promoter fragments of DNMT1, DNMT3a, and DNMT3b are listed in Table 1.

### Methylation-specific PCR (MSP) analysis

MSP based on bisulfite conversion was performed. Genomic DNA from cells was isolated using the DNA extraction kit (Qiagen, Valencia, CA, USA), and the MethylEdge™ Bisulfite Conversion System (Promega, Madison, WI, USA) was used for sodium bisulfite treatment of the genomic DNA according to the manufacturer’s protocol. The MSP primers for p21 are listed in Table 1.

### Immunocytochemistry

Cells were seeded on glass slides in 24-well plates at a density of 5000 cells/well and were fixed in 4% paraformaldehyde at room temperature (RT) for 10 min. Immunocytochemistry staining was performed using a standard immunoperoxidase staining procedure (primary antibody to Ki67 antibody 1:500 Abcam). Hematoxylin was used as a counterstain.

### Statistical analysis

All numerical data are means ± SEM. Data were analyzed with a two-tailed *t* test followed by ANOVA (GraphPad Prism, San Diego, CA). The results were considered significant at *p* < 0.05.

## Results

### Establishment of the hHFMSC cell line with ectopic expression of OCT4 (hHFMSCs^OCT4^)

**Fig. 1:**
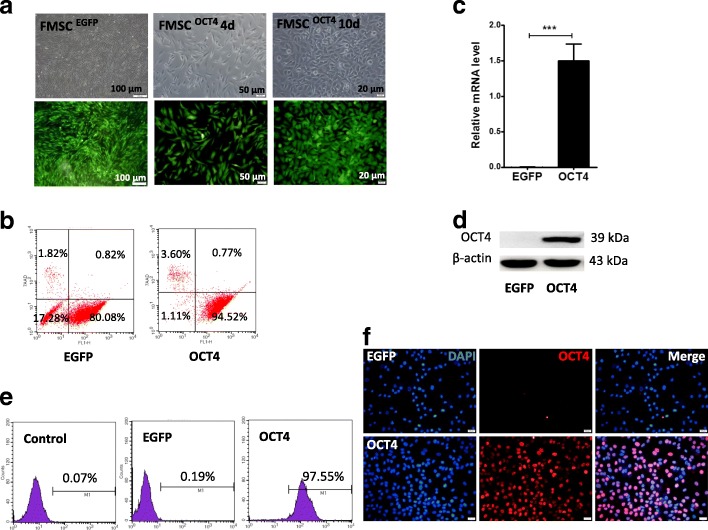
The validation of the fluorescence efficiency and the expression of OCT4 in transduced hHFMSCs. **a** The cell morphologies of transduced hHFMSCs (hHFMSCs^EGFP^ and hHFMSCs^OCT4^) were changed between 0 and 10 days after OCT4 transduction. **b** Flow cytometry assay of GFP expression in hHFMSCs after 12 days of transduction. Live cells were identified by 7AAD exclusion. qPCR (**c**), western blot (**d**), and flow cytometry (**e**) results for the expression of OCT4 in hHFMSCs^EGFP^ and hHFMSCs^OCT4^. **f** Immunofluorescence of OCT4 expression and location in hHFMSCs^EGFP^ and hHFMSCs^OCT4^ (bar, 20 μm) (EGFP, hHFMSCs^EGFP^; OCT4, hHFMSCs^OCT4^; **p < 0.01)

The proliferation, cell cycle, and differentiation potential were inhibited in hHFMSCs, and they entered into a state of replicative senescence after a certain length of cell culture (Additional file 1: Figure S1 & S2). To determine the role of OCT4 in the maintenance of hHFMSC stem cell properties, hHFMSCs were plated on Matrigel-coated dishes and infected with lentiviruses encoding OCT4 or GFP (a scramble control). Hereafter, hHFMSCs infected with scrambled lentiviruses are referred to as hHFMSCs^EGFP^, and hHFMSCs transduced with lentiviruses encoding OCT4 are referred to as hHFMSCs^OCT4^. The morphology of hHFMSCs^OCT4^ was changed after transduction compared with that of hHFMSCs^EGFP^. Cell size gradually decreased from day 0 to day 10, and original spindle-shaped cells were changed to polygonal or cobblestone-like cells (Fig. 1a). After 14 days, 80.08% of the hHFMSCs^EGFP^ cells and 94.52% of the hHFMSCs^OCT4^ cells remained GFP^+^ (Fig. 1b), suggesting a high transduction efficiency. The expression of OCT4 was further confirmed by qPCR (Fig. 1c), western blot (Fig. 1d), and flow cytometry analysis (Fig. 1e). The expression of OCT4 was markedly higher in hHFMSCs^OCT4^ than in control cells. The latter expressed a low level of endogenous OCT4. Immunofluorescence staining showed that OCT4 was located in the nuclei of the cells (Fig. 1f). Thus, we established a hHFMSC cell line, hHFMSCs^OCT4^, with ectopic expression of OCT4.

### Expression of OCT4 leads to the increased proliferative capacity, cell cycle progression, and osteogenic differentiation in hHFMSCs

**Fig. 2:**
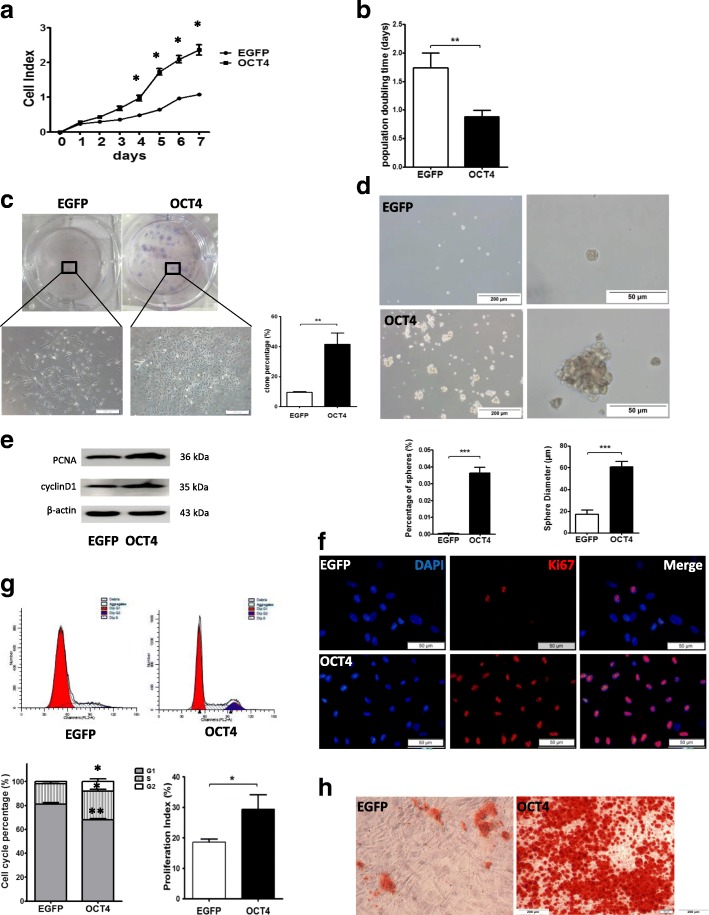
OCT4 increased proliferation capacity and differentiation potential in hHFMSCs. Cell proliferation curve (**a**) and cell population doubling time (**b**) of hHFMSCs^EGFP^ and hHFMSCs^OCT4^. **c** Clone formation assay of hHFMSCs^EGFP^ and hHFMSCs^OCT4^; the enlarged views show the difference between the two cell clones. The histogram is the formation rate for each clone (bar, 200 μm). **d** Representative sphere images and bar graph of the percentages of spheres in hHFMSCs^EGFP^ and hHFMSCs^OCT^. **e** Western blot results for the expression of proliferation-associated proteins PCNA and cyclin D1 in hHFMSCs^EGFP^ and hHFMSCs^OCT4^. **f** Immunofluorescence of proliferation-associated protein Ki67 expression and location in hHFMSCs^EGFP^ and hHFMSCs^OCT4^ (bar, 50 μm). **g** Effects of OCT4 on hHFMSC cell cycle phase distribution. The percentage of the G1, G2, and S phase in the cell cycle (left of the lower panel) and the PI (right of the lower panel) of the hHFMSCs^EGFP^ and hHFMSCs^OCT4^. “dip” is the abbreviation for diploid. **h** Osteogenic differentiation in hHFMSCs^EGFP^ and hHFMSCs^OCT4^. Calcium nodules were detected by Alizarin Red S staining (bar, 200 μm). (*p < 0.05; **p < 0.01; ***p < 0.001)

To examine the effect of OCT4 on hHFMSC proliferation, an iCELLigence cell proliferation assay was performed. The cell growth rate over a 7-day period was significantly increased in hHFMSCs^OCT4^ compared with that of hHFMSCs^EGFP^ (Fig. 2a). A shorter population doubling time was observed in hHFMSCs^OCT4^ (Fig. 2b). Colony formation was significantly higher in hHFMSCs^OCT4^ than in hHFMSCs^EGFP^. hHFMSC^EGFP^ clones exhibited scattered phenotypes, whereas hHFMSC^OCT4^ clones were relatively compacted (Fig. 2c). Sphere formation assays showed that the number and size of the spheres were significantly larger in hHFMSCs^OCT4^ (Fig. 2d). The expression of the proliferation-associated proteins PCNA, Cyclin D1, and Ki67 was upregulated in hHFMSCs^OCT4^ compared with that of hHFMSCs^EGFP^ (Fig. 2e, f).

To further clarify the influence of OCT4 on the cell cycle and proliferation, the percentage of cells in G1, G2, and S phases and the proliferative index (PI) of hHFMSCs^OCT4^ were compared with those of the control hHFMSCs^EGFP^. As shown in Fig. 2g, hHFMSCs^OCT4^ displayed a shortened G1 phase and an extended G2 and S phases (Fig. 2g). There was also a higher PI in hHFMSCs^OCT4^ (Fig. 2g).

To demonstrate the effect of OCT4 on hHFMSC differentiation potential, an osteogenic differentiation assay was performed. The results demonstrated that the osteogenic differentiation capacity was markedly increased in hHFMSCs^OCT4^ (Fig. 2h). Taken together, our data suggest that the expression of OCT4 enhances the proliferative capacity of the hHFMSCs, expedites cell cycle progression, and promotes the hHFMSCs to differentiate into osteogenic cells.

### OCT4 reverses senescence in hHFMSCs

**Fig. 3:**
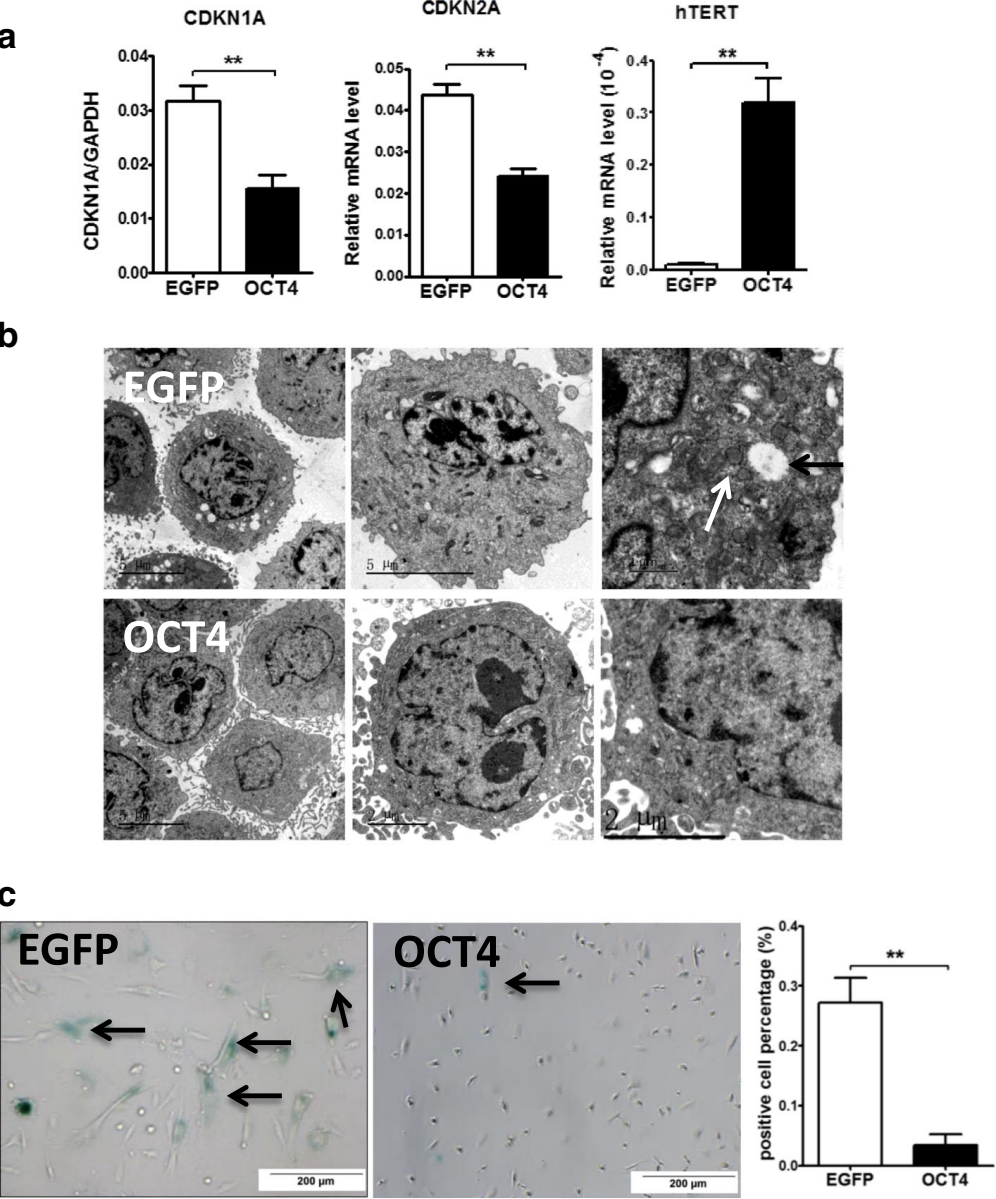
OCT4 inhibited senescence in hHFMSCs. **a** qPCR results for the expression of senescence-associated genes. CDKN1A, p21; CDKN2A, p16. **b** Effects of OCT4 on hHFMSCs under transmission electron microscopy. White arrow, dilation of the rough endoplasmic reticulum; black arrow, turgidity and vacuolization of the mitochondrion. **c** SA-β-gal staining in hHFMSCs^EGFP^ and hHFMSCs^OCT4^ (bar, 200 μm). Stained cells are indicated by arrows (**p < 0.01)

To evaluate the impact of OCT4 on hHFMSC senescence, we analyzed the expression of the senescence-associated gene human telomerase catalytic subunit (hTERT), p21 and p16, the morphology, and the SA-β-gal staining of the cells. hTERT is a catalytic subunit of the enzyme telomerase, which promotes the elongation of the telomeres in stem cells, leading to the increased lifespan of stem cells by allowing for indefinite division without the shortening of telomeres and the inhibition senescence [39, 40]. p21 coordinates cellular senescence by inducing cell cycle arrest [41]. p16 is expressed at high levels in senescing cells and plays a central role in the establishment of the senescent state [42]. qPCR showed that the expression of hTERT was upregulated, and the levels of p21 and p16 were downregulated in hHFMSCs^OCT4^ compared with hHFMSCs^EGFP^ (Fig. 3a). Through the passages of hHFMSCs^EGFP^, transmission electron microscopy showed that microvilli on the cell surface were reduced, indicating a poor proliferation capacity (Fig. 3b) [43–45]. The endoplasmic reticulum in the cells was dilated, and the mitochondria were turgid and vacuolar (Fig. 3b). The nuclei became large and irregular in some cells. These data support our notion that hHFMSCs turned senescent during culture. In contrast, in hHFMSCs^OCT4^, the cells were enriched in microvilli throughout the entire cell surface, and the nuclei were regular in shape, containing a single nucleolus or multiple nucleoli. Euchromatin was abundant in the nucleus. The cytoplasm was enriched with rough endoplasmic reticulum, mitochondria, and glycogen granules. These data indicate that the vigorous proliferative status of hHFMSCs^OCT4^ was maintained. Furthermore, the SA-β-gal staining assay revealed that hHFMSCs^OCT4^ possessed a strikingly lower level of SA-β-gal than hHFMSCs^EGFP^ (Fig. 3c). Collectively, our data suggest that OCT4 maintains the proliferative status that delays hHFMSC senescence during culture.

### Identification of differentially expressed genes in hHFMSCs^OCT4^ by next-generation sequencing

**Fig. 4:**
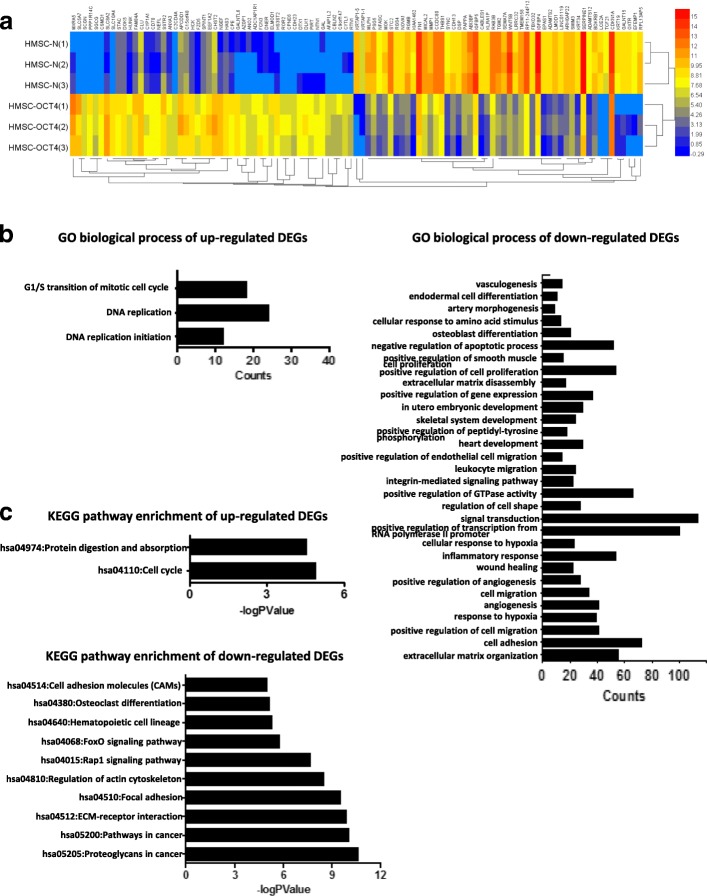
Next-generation sequencing. **a** Heat map of the top 100 differentially expressed genes (50 upregulated genes and 50 downregulated genes) and p21 (CDKN1A) between hHFMSCs^EGFP^ and hHFMSCs^OCT4^. Red, upregulation; blue, downregulation. **b** GO biological process analysis of the enrichment of differentially expressed genes between hHFMSCs^EGFP^ and hHFMSCs^OCT4^. **c** KEGG analysis of the enrichment of differentially expressed genes between hHFMSCs^EGFP^ and hHFMSCs^OCT4^ in specific pathways. GO, gene ontology; KEGG, Kyoto Encyclopedia of Genes and Genomes

To illustrate the potential molecular mechanisms by which OCT4 promotes self-renewal and reverses senescence in hHFMSCs, next-generation sequencing (NGS) was utilized. A total of 56,233 genes were analyzed, and 2466 differentially expressed genes (DEGs) were identified in hHFMSCs^OCT4^ compared with hHFMSCs^EGFP^. In the 2466 DEGs, 1181 were upregulated and 1285 were downregulated. We present 100 top DEGs, including 50 upregulated genes and 50 downregulated genes between hHFMSCs^EGFP^ and hHFMSCs^OCT4^ and p21 (CDKN1A) in Fig. 4a. The details of the DEGs (Additional file 2) and the GO (Additional file 3: Table S1) and KEGG pathway (Additional file 3: Table S2) enrichment analyses are listed in the Additional files. Briefly, GO analysis results showed that the upregulated DEGs were significantly enriched in biological processes (BPs) such as “DNA replication initiation,” “DNA replication,” and “G1/S transition of mitotic cell cycle”; the downregulated DEGs were highly enriched in BPs such as “extracellular matrix organization and cell adhesion” and “positive regulation of cell proliferation” (Fig. 4b). KEGG analysis showed that the upregulated DEGs were enriched in “cell cycle” and “protein digestion and absorption,” while the downregulated DEGs were enriched in “proteoglycans in cancer,” “pathways in cancer,” “ECM-receptor interaction,” “PI3K-Akt signaling pathway,” “focal adhesion,” and “regulation of actin cytoskeleton” (Fig. 4c). Thus, we conclude that OCT4 may maintain the self-renewal and reverse the senescence of hHFMSCs by regulating the comprehensive signaling pathways in cell biological processes. As p21 has been known to play a negative role in cell cycle signaling, we hypothesize that OCT4 maintains self-renewal and reverses senescence through p21.

### The overexpression of p21 abolishes the effects of OCT4 on hHFMSCs

**Fig. 5:**
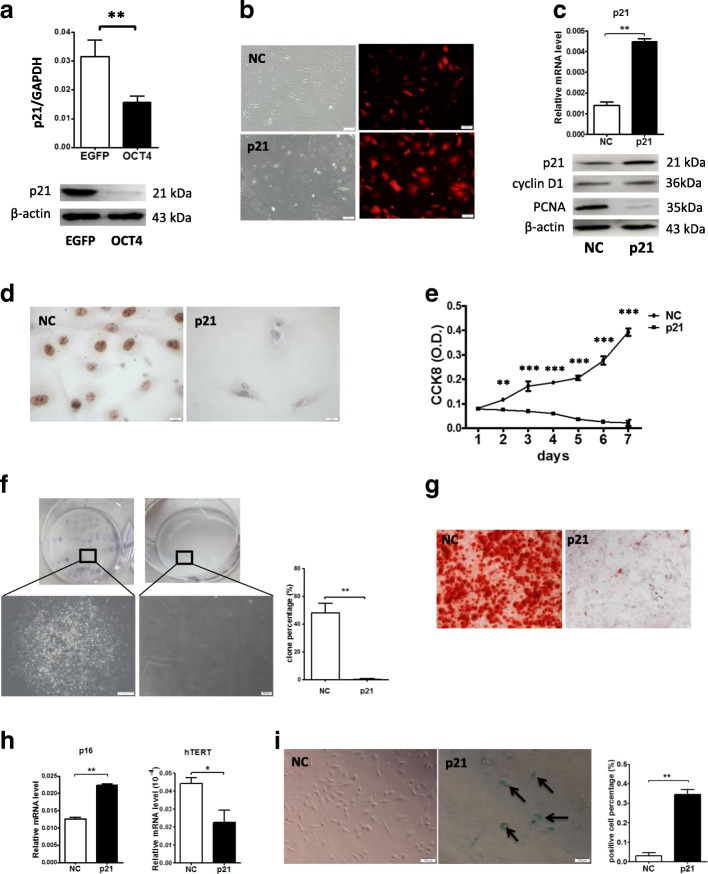
The overexpression of p21 abolished the effect of OCT4 in hHFMSCs. **a** qPCR and western blot of the expression of p21 in hHFMSCs^EGFP^ and hHFMSCs^OCT4^. **b** The overexpression of p21 in hHFMSCs^OCT4^ (named NC and p21; bar, 100 μm). **c** qPCR and western blot after overexpressing p21. **d** Immunocytochemistry of Ki67 after overexpressing p21 (bar, 20 μm). Cell proliferation curve (**e**) and clone formation assay (**f**) after overexpressing p21; the enlarged views show the difference between the two cell clones. The histogram is the formation rates of the clones (bar, 200 μm). **g** Osteogenic differentiation after overexpressing p21 in hHFMSCs^OCT4^. Calcium nodules were detected by Alizarin Red S staining (bar, 200 μm). **h** qPCR results for the expression of senescence-associated genes. **i** SA-β-gal staining after overexpressing p21 in hHFMSCs^OCT4^ (bar, 200 μm). Stained cells are indicated by arrows (*p < 0.05; **p < 0.01; ***p < 0.001)

p21, which controls the cell entry into the cell cycle, was selected from NGS, as p21 was significantly downregulated in NGS (fold change = 2.807691, *p* = 0.000751684). The downregulation of p21 was verified by qRT-PCR and western blot (Fig. 5a). As a gene negatively associated with self-renewal and a senescence marker, p21 plays critical roles in the regulation of the G1/S transition. Thus, we speculate that OCT4 may assist the hHFMSCs to regain stemness through the downregulation of p21. To test this assumption, we overexpressed p21 in hHFMSCs^OCT4^. As a result, cells with p21 overexpression became enlarged and flat (Fig. 5b). Following p21 overexpression, PCNA and Ki67 were downregulated, but the expression of cyclin D1 was not influenced in hHFMSCs^OCT4^ (Fig. 5c, d). The proliferation and colony formation of the cells were suppressed, and the osteogenic differentiation capacity was inhibited with p21 overexpression compared with that of the control (Fig. 5e–g). Moreover, we observed that a gene positively associated with senescence, p16, was also upregulated, and a gene negatively associated with senescence, hTERT, was downregulated (Fig. 5h). Additionally, SA-β-gal staining was increased when p21 was overexpressed in hHFMSCs^OCT4^ (Fig. 5i). Taken together, these data suggest that p21 plays a critical role in the suppression of the stem cell properties of hHFMSCs^OCT4^.

### Overexpression of OCT4 promotes the transcription of DNMTs and elevates p21 methylation in hHFMSCs

**Fig. 6:**
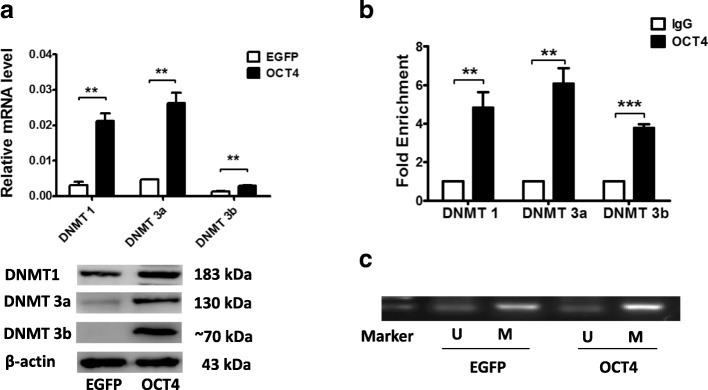
OCT4 regulates p21 through DNMTs. **a** qPCR and western blot analyses of the expression levels of DNMT1, DNMT3a, and DNMT3b in hHFMSCs^EGFP^ and hHFMSCs^OCT4^. **b** ChIP-qPCR of the transcription factor OCT4 and the genes DNMT1, DNMT3a, and DNMT3b. **c** MSP of p21 in hHFMSCs^EGFP^ and hHFMSCs^OCT4^ (**p < 0.01; ***p < 0.001)

As the transcriptional level of p21 is regulated by DNA methylation [27], the expression of DNMTs was quantified in hHFMSCs^EGFP^ and hHFMSCs^OCT4^. DNMT1, DNMT3a, and DNMT3b were upregulated in hHFMSCs^OCT4^ at both the mRNA and protein levels (Fig. 6a). To detect whether the transcriptional factor OCT4 upregulated DNMTs by binding to their promoter regions in hHFMSCs, a ChIP assay was performed (Fig. 6b). This result confirmed that OCT4 interacts with the promoter regions of DNMT1, DNMT3a, and DNMT3b, which may promote the transcription of DNMTs. To illustrate the methylation state of the p21 promoter region, we performed an MSP assay. As shown in Fig. 6c, the methylation level of the p21 promoter region was markedly higher, and the amount of the promoter that was not methylated was lower in hHFMSCs^OCT4^ than in hHFMSCs^EGFP^. Taken together, our data support our notion that DNMTs are upregulated in OCT4-overexpressing hHFMSCs, which may contribute to the elevated methylation and reduced expression of p21 in the cells.

### Inhibition of DNMTs abrogates OCT4-supported stemness in hHFMSCs

**Fig. 7:**
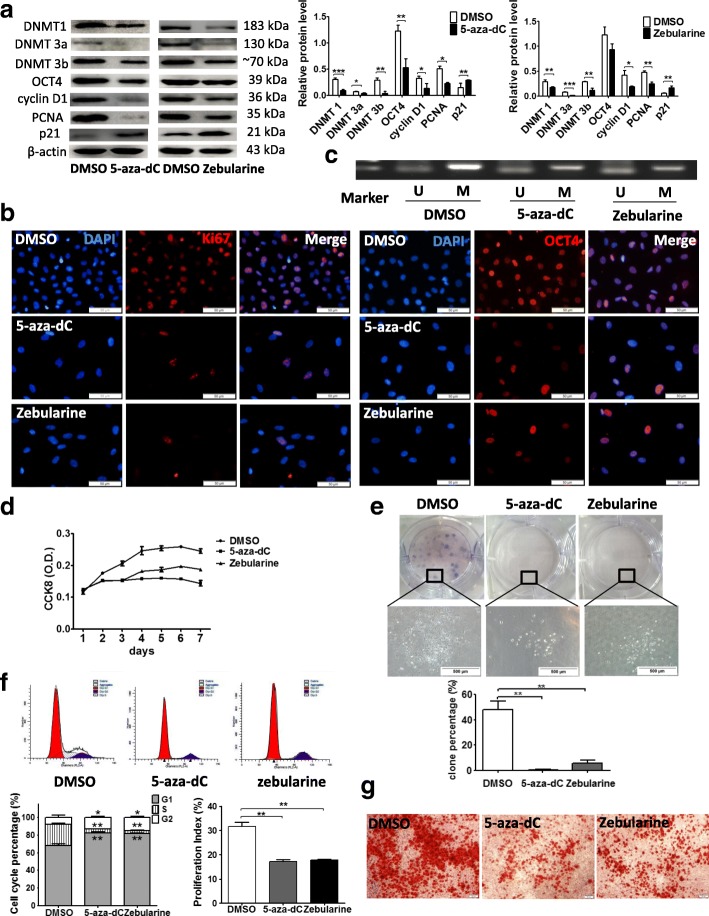
DNMT inhibition decreased OCT4-increased proliferation capacity and differentiation potential in hHFMSCs. **a** The impact of 5-aza-dC (100 μM, 72 h) and zebularine (100 μM, 48 h) treatment on the expression of DNMTs, p21, OCT4, PCNA, and cyclin D1. **b** Immunofluorescence of proliferation-associated protein Ki67 (left panels) and OCT4 (right panels) expression and location after DNMT inhibition (bar, 50 μm). **c** MSP of p21 in hHFMSCs^OCT4^ treated with DMSO, 5-aza-dC, or zebularine. Cell proliferation curve (**d**) and clone formation assay (**e**) after DNMT inhibition; and the enlarged views showed the difference between DMSO and DNMT inhibitor-treated hHFMSCs^OCT4^. The histogram is the formation rate of the three clones. **f** Effects of DNMT inhibition on hHFMSC cell cycle phase distribution. The percentage of the G1, G2, and S phases in the cell cycle (left of the lower panel) and the PI (right of the lower panel) after DNMT inhibition. “dip” is the abbreviation for diploid. **g** Osteogenic differentiation after DNMT inhibition. Calcium nodules were detected by Alizarin Red S staining (bar, 50 μm). (*p < 0.05; **p < 0.01)

To further characterize the role of DNMTs in the stem cell phenotypes maintained by OCT4 in hHFMSCs, the DNMT inhibitor 5-aza-dC and zebularine were applied to hHFMSCs^OCT4^. The administration of 5-aza-dC and zebularine led to a dosage-dependent and time course-associated suppression of DNMT activities (Additional file 1: Figures S3 and S4). More importantly, following the inhibition of DNMTs, p21 was strikingly elevated along with the downregulation of proliferation-associated proteins cyclin D1, PCNA, and Ki67 (Fig. 7a, b). Interestingly, the inhibition of DNMTs with 5-aza-dC modestly downregulated the expression of OCT4 (Fig. 7a), although the nuclear location of OCT4 was not affected by the treatment (Fig. 7b). In addition, we observed that the methylation of the p21 promoter region was downregulated and that the amount of the promoter that was not methylated was upregulated when DNMTs were inhibited in hHFMSCs^OCT4^ (Fig. 7c), supporting our conclusion that the downregulation of p21 in hHFMSCs^OCT4^ was mediated by DNMTs.

**Fig. 8:**
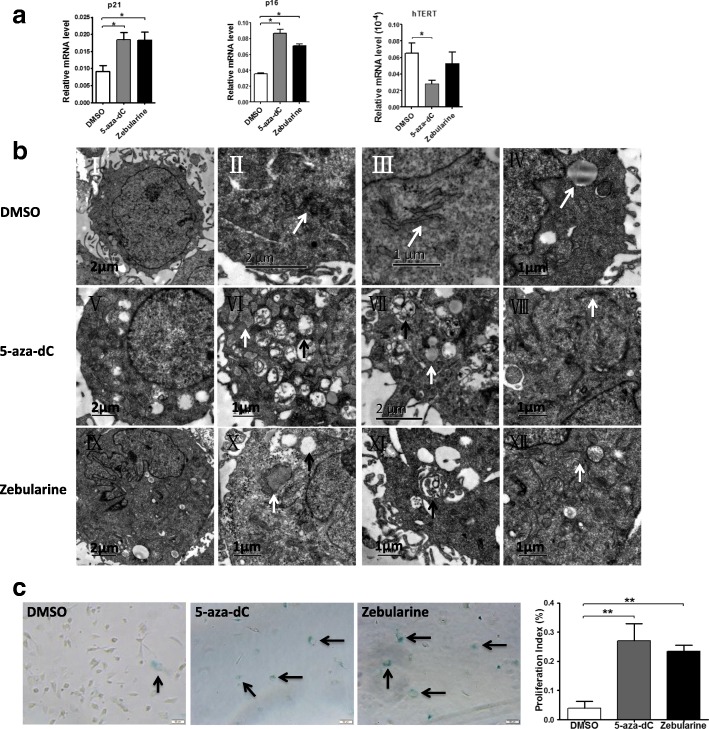
DNMT inhibition promoted senescence in hHFMSCs. **a** qPCR results for the expression of senescence-associated genes. **b** Effects of DNMT inhibition on hHFMSCs under transmission electron microscopy. (I) Rich microvilli in the cell surface, regularly shaped nuclei, and abundant euchromatin in the nucleus. (II) Normal mitochondria. (III) The cytoplasm rich in rough endoplasmic reticulum. (IV) Some cells containing lipid drops. (V, IX) The microvilli in the cell surface were almost completely removed, and some of the nuclei are irregular in shape. (VI, X) The dilation of the rough endoplasmic reticulum (white arrow) and the turgidity and vacuolization of the mitochondrion (black arrow). (VII, XI) The secondary lysosomes (black arrow) and lipid drops (white arrow) in some cells. (VIII, XII) The contraction of the Golgi body. **c** SA-β-gal staining after DNMT inhibition (bar, 50 μm); stained cells are indicated by arrows. (*p < 0.05; **p < 0.01)

As expected, the inhibition of DNMTs led to a reduced proliferative rate (Fig. 7d) and decreased colony formation capacity (Fig. 7e) in hHFMSCs^OCT4^. These cells also displayed a G1 phase arrest and a low PI (Fig. 7f). Additionally, the osteogenic differentiation capacity of hHFMSCs^OCT4^ was also blocked due to the exposure to DNMT inhibitors (Fig. 7g). These data suggested that DNMT inhibition suppressed the stem cell properties in hHFMSCs^OCT4^. Moreover, genes positively associated with senescence, p21 and p16, were upregulated, and a gene negatively associated with senescence, hTERT, was downregulated by DNMT inhibition in the cells (Fig. 8a). Electron microscopy showed that the cells manifested in a senescent state after DNMT inhibition (Fig. 8b) and that the level of positive SA-β-gal staining was high when DNMTs were inhibited in hHFMSCs^OCT4^ (Fig. 8c). These data suggest that DNMT inhibition promotes hHFMSC senescence.

Thus, we demonstrated that OCT4 regulated hHFMSC self-renewal and senescence through downregulating p21 by DNMTs.

## Discussion

It was reported that the pluripotency gene OCT4 plays a pivotal role in the maintenance of stem cell properties by regulating self-renewal and senescence. Here, we report that OCT4 maintained self-renewal and reversed senescence in hHFMSCs. After the ectopic expressing of OCT4, we found that OCT4 was localized in the nucleus of hHFMSCs^OCT4^. OCT4 promoted cell proliferation and osteogenic differentiation and reversed senescence in hHFMSCs. NGS showed that p21 was downregulated in hHFMSCs^OCT4^. KEGG pathway analysis revealed enrichment in the alteration of cell cycle-associated genes. We further demonstrated that OCT4 enhanced the expression of DNMT1, DNMT3a, and DNMT3b through binding to these DNMT promoter regions. Additionally, the overexpression of p21 or the inhibition of DNMTs abolished the functional role of OCT4 in the maintenance of the stemness of hHFMSCs. Collectively, our data demonstrate that OCT4 was essential for maintaining self-renewal properties and for reversing senescence in hHFMSCs. OCT4 promoted the transcriptional activation of DNMTs and hence elevated the methylation of p21, leading to cell cycle progression and suppression of senescence in hHFMSCs.

In previous studies [46, 47], the overexpression or silencing of OCT4 showed similar results, demonstrating that OCT4 can promote cell proliferation capacity. It was reported that OCT4 binds to the promoter region of miR-302a and hence promotes an increase of S phase cells and a decrease of G1 phase cells through cyclin D1 in hESCs [48]. The overexpression of OCT4 and SOX2 could also increase S phase and decrease G1 phase probably through the upregulation of cyclin D1 in hATMSCs [49]. Similarly, we found that the expression of OCT4 led to an increase in S and G2 phase cells as well as a decrease of G1 phase cells and the upregulation of cyclin D1. Consequently, OCT4 participates in the regulation of the cell cycle, which is a critical process for determining cell proliferation and senescence [50] and is vital to maintaining stem cell properties [51]. In addition, an extended G1 phase may be detrimental to stem cell identity [52]. In the current study, we found that the overexpression of OCT4 improved the osteogenic differentiation capacity, which is consistent with previous studies [53–55]. Moreover, similar to previous studies [18, 56], we found that OCT4 prevented hHFMSCs from entering a state of senescence, as evidenced by the expression of senescence-associated markers, morphological alterations in ultrastructure, and SA-β-gal staining.

OCT4 has been reported previously to drive the expression of a number of pluripotent-specific genes [57]. In the present study, we identified genes regulated by OCT4 using NGS in hHFMSCs. We also determined the enrichment of signaling pathways associated with OCT4 expression in hHFMSCs using GO and KEGG analyses. As a result, the cell cycle pathway was identified. The cell cycle plays a pivotal role in the OCT4-mediated maintenance of the stem cell state. p21, a cyclin-dependent kinase inhibitor, promotes cell cycle arrest. NGS data showed that p21 was significantly downregulated in hHFMSCs^OCT4^. Previous reports demonstrated that p21 has a negative impact on stem cell functions [58, 59]. Therefore, p21 seems to be an important gene in OCT4-maintained hHFMSC stemness.

Our current data showed that the overexpression of p21 abolished the functional roles of OCT4 in hHFMSCs, except for the expression of cyclin D1. Cyclin D1, when expressed, associates with a cyclin-dependent kinase (CDK) 4 or 6, and this complex phosphorylates and activates genes whose products regulate the G1/S transition of the cell cycle [23]. The activity of the cyclin D1/CDK complex is also regulated by p21 [22, 60]. Thus, p21 inhibits the activity of the cyclin D1 complex instead of its expression. The upregulation of cyclin D1 in hHFMSCs^OCT4^ and the downregulation of the gene after DNMT treatment may be due to alternative signals. It would be interesting to pursue the exact mechanism of cyclin D1 regulation in hHFMSCs^OCT4^ in the future.

DNA methylation is essential during development, resulting in the silencing of early embryonic genes and the activation of the lineage-specific genes that drive cell differentiation [61]. Tissue-specific genes are upregulated in methylation-deficient mESCs [62]. Moreover, methylation is activated in induced pluripotent stem cells (iPSCs) from parental mouse embryonic fibroblasts [63]. Hence, DNMTs play a distinct role in maintaining stem cell properties. DNA methylation changes in aged tissues and cells [64, 65], regulates the expression of a certain portion of genes, and partly contributes to the introduction and establishment of senescence [66]. Meanwhile, DNMTs are downregulated along with continuous passage. The current data showed that DNMT inhibition could abolish the functions of OCT4 in hHFMSCs and that OCT4 enhanced the expression of DNMTs through binding to their promoter region. These results support the notion that OCT4 maintains hHFMSC properties through the DNMT-regulated downstream gene p21.

## Conclusions

In this study, we focused on the role of OCT4 in maintaining hHFMSC properties and found that OCT4 was essential for maintaining self-renewal properties and reversing senescence in hHFMSCs. The mechanism underlying this is that OCT4 could suppress p21, the downstream stem cell property-associated gene, through upregulating DNMTs. These results provide a theoretical basis for hHFMSCs in regenerative medicine and may be beneficial for personalized cell therapy.

## Additional files

Additional file 1: **Figure S1.** OCT4 increased proliferation capacity and differentiation potential in hHFMSCs. Cell proliferation curve (a) and Clone formation assay (b) of hHFMSCs-P5 and hHFMSCs-P15, and the enlarged views showed the difference between the two cell clones (bar, 200 μm). (c) Immunofluorescence of proliferation associated protein Ki67 expression and location in hHFMSCs-P5 and hHFMSCs-P15 (bar, 50 μm). (d) Cell cycle assay. The proliferation index (left of the lower panel), and the percentage of G1-, G2-, and S phase in the cell cycle (right of the lower panel) in hHFMSCs-P5 and hHFMSCs-P15. Adipogenic (e) and osteogenic (f) differentiation (bar, 500 μm). (g) qPCR results for the expression of senescence-associated gene. (h) SA-β-gal staining in hHFMSCs-P5 and hHFMSCs-P15 (bar, 500 μm), stained cells were indicated by arrows (*p < 0.05; **p < 0.01). **Figure S2.** qPCR (a) and western blot (b) results in hHFMSCs-P5 and hHFMSCs-P15 (*p < 0.05; **p < 0.01). **Figure S3.** 5-aza-dC and zebularine downregulated DNMT1 expression in a dose-dependent manner (*p < 0.05; **p < 0.01). **Figure S4.** 5-aza-dC and zebularine downregulated DNMT1 expression in a time-dependent manner (**p < 0.01). (DOCX 1448 kb)

Additional file 2: The details of the DEGs. (XLSX 161 kb)

Additional file 3: **Table S1.** Gene ontology analysis of differentially expressed genes. **Table S2.** KEGG pathway analysis of differentially expressed genes associated with colorectal cancer. (DOCX 38 kb)

## Abbreviations

7AAD: 7-Amino actinomycin
bFGF: Basic fibroblast growth factor
CDK: Cyclin-dependent kinase
ChIP: Chromatin immunoprecipitation
DEGs: Differentially expressed genes
DMSO: Dimethyl sulfoxide
DNMT1: DNA methyltransferase 1
DNMT3a: DNA methyltransferase 3 alpha
DNMT3b: DNA methyltransferase 3 beta
DNMTs: DNA methyltransferases
ESCs: Embryonic stem cells
FBS: Fetal bovine serum
hHFMSCs: Human hair follicle mesenchymal stem cells
hHFMSCs^EGFP^: hHFMSCs transfected with scrambled lentivirus
hHFMSCs^OCT4^: hHFMSCs transfected with lentivirus encoding the OCT4
hTERT: Human telomerase catalytic subunit
iPSCs: Induced pluripotent stem cells
KEGG: Kyoto Encyclopedia of Genes and Genomes
MSCs: Mesenchymal stem cells
MSP: Methylation-specific PCR
NGS: Next-generation sequencing
PCNA: Proliferating cell nuclear antigen
PI: Proliferation index
SA-β-gal: Senescence-associated β-galactosidase
TFs: Transcription factors
