# Supplementary material for: OCT4 maintains self-renewal and reverses senescence in human hair follicle mesenchymal stem cells through the downregulation of p21 by DNA methyltransferases
Source: Stem Cell Res Ther. 2019 Jan 15;10:28. doi: 10.1186/s13287-018-1120-x (PMC6334457; doi:10.1186/s13287-018-1120-x)
Supplement: Supplementary file 1 — Figure S1. OCT4 increased proliferation capacity and differentiation potential in hHFMSCs. Cell proliferation curve (a) and Clone formation assay (b) of hHFMSCs-P5 and hHFMSCs-P15, and the enlarged views showed the difference between the two cell clones (bar, 200 μm). (c) Immunofluorescence of proliferation associated protein Ki67 expression and location in hHFMSCs-P5 and hHFMSCs-P15 (bar, 50 μm). (d) Cell cycle assay. The proliferation index (left of the lower panel), and the percentage of G1-, G2-, and S phase in the cell cycle (right of the lower panel) in hHFMSCs-P5 and hHFMSCs-P15. Adipogenic (e) and osteogenic (f) differentiation (bar, 500 μm). (g) qPCR results for the expression of senescence-associated gene. (h) SA-β-gal staining in hHFMSCs-P5 and hHFMSCs-P15 (bar, 500 μm), stained cells were indicated by arrows (*p < 0.05; **p < 0.01). Figure S2. qPCR (a) and western blot (b) results in hHFMSCs-P5 and hHFMSCs-P15 (*p < 0.05; **p < 0.01). Figure S3. 5-aza-dC and zebularine downregulated DNMT1 expression in a dose-dependent manner (*p < 0.05; **p < 0.01). Figure S4. 5-aza-dC and zebularine downregulated DNMT1 expression in a time-dependent manner (**p < 0.01). (DOCX 1448 kb) [file 13287_2018_1120_MOESM1_ESM.docx]

**Supplementary figures**

**
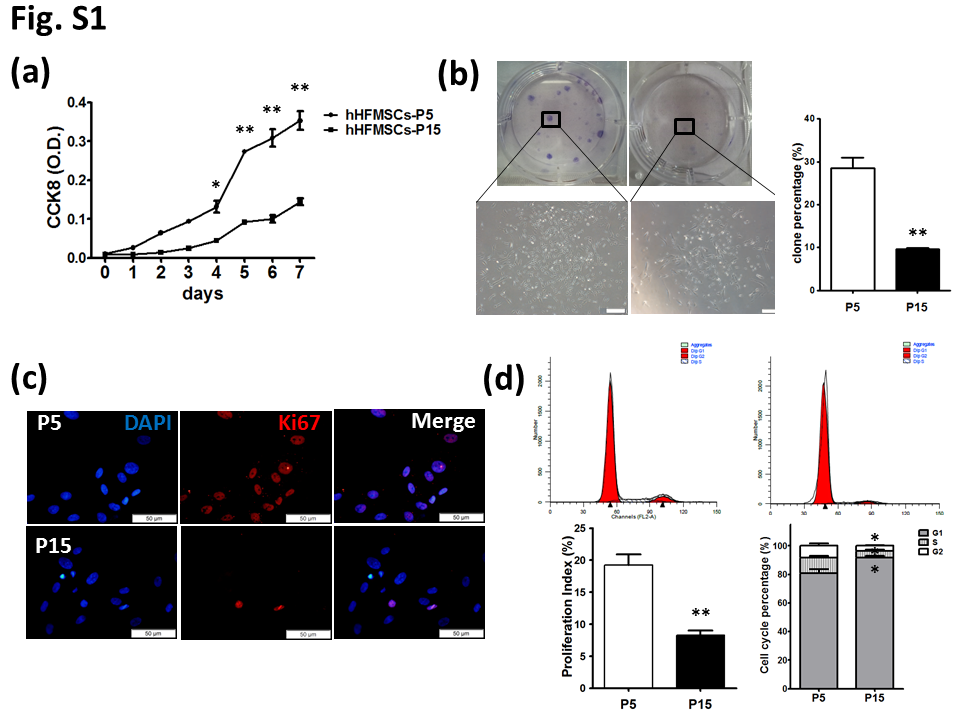
**

**
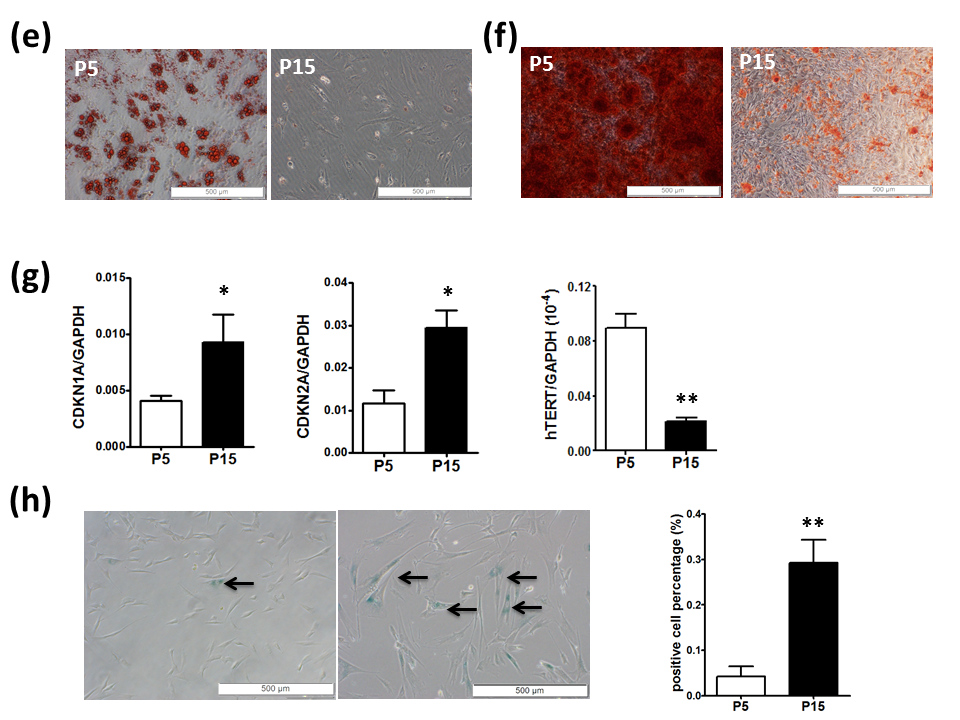
**

**Fig. S1 OCT4 increased proliferation capacity and differentiation potential in hHFMSCs**

Cell proliferation curve (a) and Clone formation assay (b) of hHFMSCs-P5 and hHFMSCs-P15, and the enlarged views showed the difference between the two cell clones (bar, 200 μm). (c) Immunofluorescence of proliferation associated protein Ki67 expression and location in hHFMSCs-P5 and hHFMSCs-P15 (bar, 50 μm). (d) Cell cycle assay. The proliferation index (left of the lower panel), and the percentage of G1-, G2-, and S-phase in cell cycle (right of the lower panel) in hHFMSCs-P5 and hHFMSCs-P15. Adipogenic (e) and osteogenic (f) differentiation (bar, 500 μm). (g) qPCR results for the expression of senescence associated gene. (h) SA-β-gal staining in hHFMSCs-P5 and hHFMSCs-P15 (bar, 500 μm), stained cells were indicated by arrows (*, *p* < 0.05; **, *p* < 0.01).


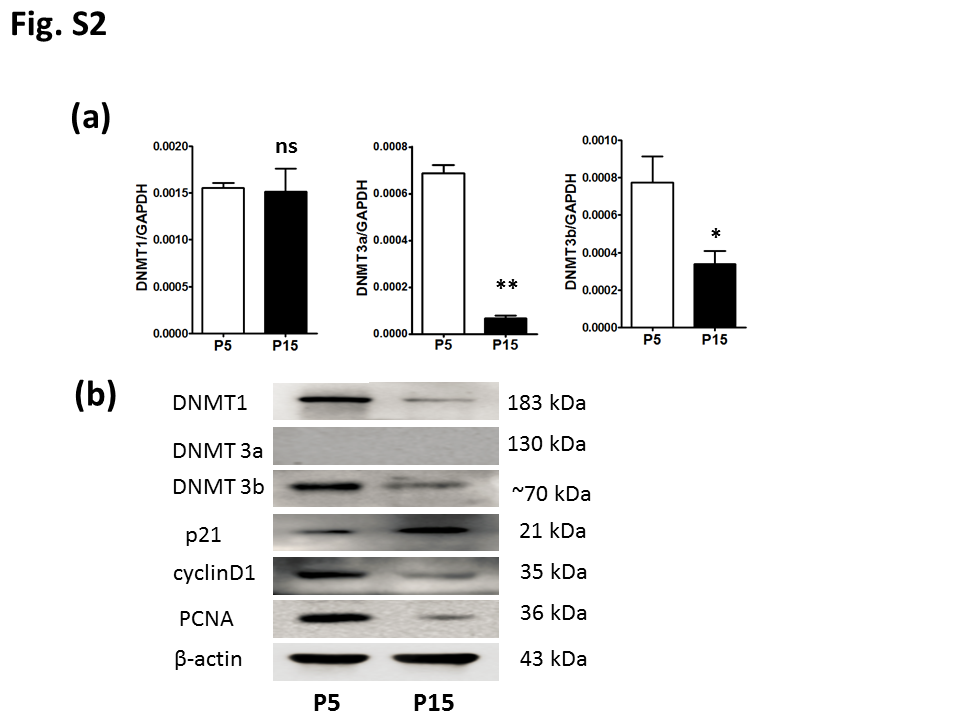


**Fig. S2** qPCR (a) and Western blot (b) results in hHFMSCs-P5 and hHFMSCs-P15 (*, *p* < 0.05; **, *p* < 0.01).


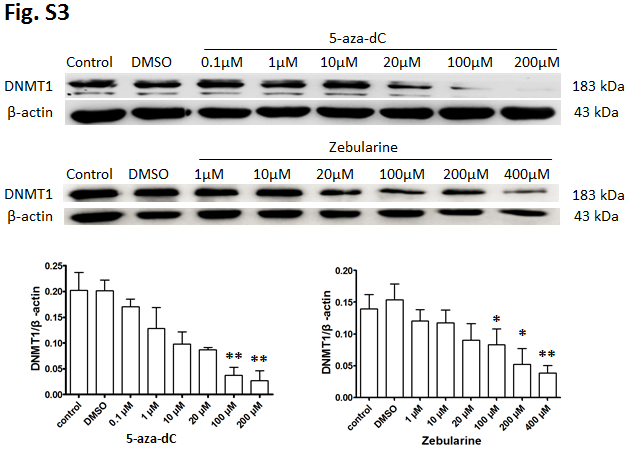


**Fig. S3** 5-aza-dC and zebularine downregulated DNMT1 expression in a dose-dependent manner (*, *p* < 0.05; **, *p* < 0.01)


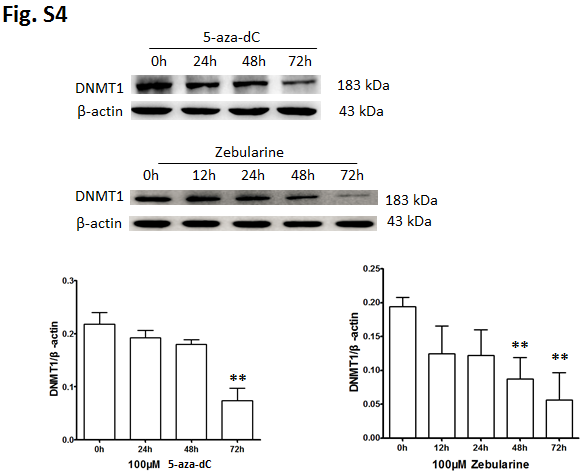


**Fig. S4** 5-aza-dC and zebularine downregulated DNMT1 expression in a time-dependent manner (**, *p* < 0.01)
