# Supplementary material for: OCT4 maintains self-renewal and reverses senescence in human hair follicle mesenchymal stem cells through the downregulation of p21 by DNA methyltransferases
Source: Stem Cell Res Ther. 2019 Jan 15;10:28. doi: 10.1186/s13287-018-1120-x (PMC6334457; doi:10.1186/s13287-018-1120-x)
Supplement: Supplementary file 3 — Table S1. Gene ontology analysis of differentially expressed genes. Table S2. KEGG pathway analysis of differentially expressed genes associated with colorectal cancer. (DOCX 38 kb) [file 13287_2018_1120_MOESM3_ESM.docx]

**Table S1 Gene ontology analysis of differentially expressed genes**

| expression | Category | Term | Count | genes | FDR |
| --- | --- | --- | --- | --- | --- |
| Up-regulated DEGs | GOTERM_BP_DIRECT | GO:0006270~DNA replication initiation | 12 | PRIM1, CDC6, CDC45, MCM7, POLA1, MCM2, MCM3, MCM10, MCM4, ORC1, MCM5, MCM6 | 3.06E-04 |
|  | GOTERM_BP_DIRECT | GO:0006260~DNA replication | 24 | CLSPN, CDC6, GINS2, GINS3, NASP, POLA1, KIAA0101, MCM2, CDC25C, MCM10, MCM3, MCM4, CDC25A, MCM5, MCM6, CDC45, RFC3, MCM7, TIMELESS, POLE3, RRM2, CHTF18, ORC1, DUT | 0.003084 |
|  | GOTERM_BP_DIRECT | GO:0000082~G1/S transition of mitotic cell cycle | 18 | CDC6, POLA1, PKMYT1, MCM2, MCM10, MCM3, MCM4, CDC25A, MCM5, MCM6, PRIM1, CDC45, DHFR, MCM7, RRM2, RANBP1, CDCA5, ORC1 | 0.014214 |
|  | GOTERM_CC_DIRECT | GO:0014069~postsynaptic density | 25 | DNM3, ANKS1B, SYNDIG1, LYN, KCNAB2, TANC1, STRN, SPOCK1, PCLO, ITPR1, EPB41L3, DAB1, CNIH2, GRIA1, SIPA1L1, NTRK2, MAPK8IP2, NEFH, DNAJC6, LRP8, NRGN, AXIN2, ADD2, GAP43, NEURL1 | 0.010578 |
|  | GOTERM_CC_DIRECT | GO:0005578~proteinaceous extracellular matrix | 31 | ASPN, AMTN, ADAMTS13, ADAMTSL3, SPOCK1, WNT2, COL9A3, HPSE, COMP, GPC6, TFF3, SERPINA1, COL11A1, DPT, COL10A1, BMP4, FLRT1, TNXB, IL1RL1, OLFML2B, CILP, COL15A1, COL5A2, SLIT1, SLIT2, COL14A1, WNT11, WNT9A, ADAMTS3, COL24A1, NCAN | 0.01855 |
|  | GOTERM_CC_DIRECT | GO:0042555~MCM complex | 6 | MCM7, MCM2, MCM3, MCM4, MCM5, MCM6 | 0.037655 |
| Down-regulated DEGs | GOTERM_BP_DIRECT | GO:0030198~extracellular matrix organization | 55 | PXDN, NFKB2, TNFRSF11B, CD44, FOXF1, SERPINE1, TGFBI, LOX, LOXL1, FGF2, CYR61, RECK, ICAM1, F11R, OLFML2A, BGN, COL1A2, VCAN, LAMC2, LAMC1, COL1A1, JAM2, MFAP5, LUM, ITGA11, ITGA10, ITGB5, ITGB2, DCN, ITGB3, ABI3BP, ERO1A, COL6A3, COL6A2, BCL3, COL6A1, FBN2, THBS1, COL8A2, FN1, B4GALT1, COL4A2, COL4A1, HSPG2, CCDC80, ITGA2, ITGA3, NID2, LAMA1, FBLN1, ITGA6, ITGA5, ITGA8, ITGA7, APBB2 | 5.31E-19 |
|  | GOTERM_BP_DIRECT | GO:0007155~cell adhesion | 72 | NRP2, OPCML, TLN2, BCAR1, EDIL3, CXCL12, WISP2, KIAA1462, SRPX, CD44, CTGF, TGFBI, CNTNAP1, SPON2, LOXL2, CYR61, ICAM1, F11R, MAGI1, EFNB1, MFGE8, PTPRU, CERCAM, SIRPA, VCAN, LAMC2, CNTN3, LAMC1, TGFB1I1, COL1A1, ITGA11, ITGA10, ITGB5, IL32, ITGB2, PCDHGC3, CDH2, ITGB3, RGMB, LPXN, FAT1, COL6A3, COL6A2, COL6A1, CD4, THBS1, APBA1, FN1, B4GALT1, ADAM23, PODXL, EFS, ITGA2, ITGA3, NID2, MCAM, EMILIN2, PRPH2, ADGRG1, GAS6, LAMA1, CDH13, CDH15, ITGA6, ITGA5, ITGA8, ITGA7, ABL2, THEMIS2, ENG, NTM, FEZ1 | 1.35E-10 |
|  | GOTERM_BP_DIRECT | GO:0030335~positive regulation of cell migration | 41 | CCL3, WNT5B, CSF1, BCAR1, EDN1, CCL26, DAB2, ZNF703, SEMA7A, SEMA3F, FOXF1, NUMB, PDGFC, HSPA5, FGF1, THBS1, CYR61, EGFR, PODXL, PIK3CD, SMAD3, MCAM, SNAI1, CDH13, ITGA6, SEMA4F, ITGA5, F3, RRAS2, CD274, VEGFA, CEMIP, PDGFRA, HBEGF, TCAF2, HAS2, LAMC2, COL1A1, PLAU, MYLK, F2R | 9.15E-10 |
|  | GOTERM_BP_DIRECT | GO:0001666~response to hypoxia | 39 | PPARA, PAM, LDHA, ACVRL1, ARNT2, PML, BNIP3, PDLIM1, EGLN1, MMP2, CXCL12, TGFB2, CITED2, SLC11A2, PLOD1, MYOCD, ANG, PLOD2, TEK, CAMK2D, THBS1, LOXL2, DPP4, ANGPTL4, PLAT, EPAS1, TGFBR2, ITGA2, SMAD3, CAPN2, AJUBA, LEP, HIF1A, CA9, ADM, PENK, VEGFA, ENG, PLAU | 2.07E-09 |
|  | GOTERM_BP_DIRECT | GO:0001525~angiogenesis | 41 | NRP2, FGFR1, ACVRL1, TNFRSF12A, CSPG4, CXCL8, ANPEP, MMP2, GJA5, TGFB2, ARHGAP22, ANG, CTGF, TEK, SERPINE1, TGFBI, FGF1, COL8A2, ANGPTL4, FN1, FZD8, FMNL3, COL4A2, EPAS1, HSPG2, MFGE8, ARHGAP24, MCAM, MYH9, ECM1, ADGRG1, PRKD1, LEP, HIF1A, SRPX2, ITGA5, CLIC4, VEGFA, ECSCR, HIF3A, ADAM15 | 5.02E-07 |
|  | GOTERM_BP_DIRECT | GO:0016477~cell migration | 34 | FGFR1, CTHRC1, CD248, BCAR1, PEAK1, CSPG4, BDKRB1, ITGB3, CDH2, SDC4, TGFB2, ANG, CTGF, FAT1, ADAMTS12, THBS1, PSTPIP2, FMNL3, PLXNB1, PODXL, PSG2, PALLD, SNAI1, ADGRG1, GAS6, EPHA2, TNS3, SDC1, NDEL1, FYN, FOXC1, LAMC1, ENG, ABL2 | 3.14E-06 |
|  | GOTERM_BP_DIRECT | GO:0045766~positive regulation of angiogenesis | 27 | WNT5A, ACVRL1, C3, CXCL8, ITGB2, GREM1, PTGIS, GATA6, CCBE1, SERPINE1, TEK, IL1B, RRAS, FGF1, THBS1, FGF2, DDAH1, ANGPTL4, TGFBR2, ECM1, PRKD1, HIF1A, ADM, F3, HIPK2, VEGFA, ENG | 4.55E-06 |
|  | GOTERM_BP_DIRECT | GO:0042060~wound healing | 22 | WNT5A, DCBLD2, EGFR, PPARA, IL6, WNT5B, TGFBR2, SMAD3, DCN, ITGB3, SDC4, TPM1, TGFB2, MAP3K5, SDC1, SERPINB2, PDGFRA, DSP, LOX, FGF2, ENG, FN1 | 9.03E-06 |
|  | GOTERM_BP_DIRECT | GO:0006954~inflammatory response | 53 | TNFRSF6B, CXCL1, TPST1, TNFRSF21, CCL3, CXCL5, C3, ADORA2A, CSF1, PTGS1, CXCL8, BDKRB1, ITGB2, NFKB2, BDKRB2, IL34, CXCL12, CCL26, TNFRSF11B, PTGIR, TNFRSF1B, SEMA7A, TICAM2, IL1B, MGLL, BCL6, PTX3, THBS1, TNIP1, IRAK2, F11R, IL6, PTGER2, CEBPB, LY96, PIK3CD, RELB, AXL, PTGFR, ECM1, NLRP1, EPHA2, PRKD1, HDAC5, GGT5, SDC1, TNFRSF10D, AOX1, RIPK2, TNFAIP3, THEMIS2, IGFBP4, F2R | 2.30E-05 |
|  | GOTERM_BP_DIRECT | GO:0071456~cellular response to hypoxia | 23 | VASN, ICAM1, SLC8A1, STC2, EPAS1, EDN1, BNIP3, PINK1, CPEB1, ANKRD1, FOXO3, KCNK2, ZFP36L1, FMN2, PTGIS, HIF1A, SFRP1, ERO1A, GATA6, HIPK2, VEGFA, STC1, MGARP | 5.87E-05 |
|  | GOTERM_BP_DIRECT | GO:0045944~positive regulation of transcription from RNA polymerase II promoter | 100 | EDN1, ARNT2, FSTL3, ANKRD1, FOXO3, CITED4, GDNF, IL11, CITED2, FLI1, MYOCD, GATA6, FOXF1, SERPINE1, IL1B, CREB3L1, PITX1, EGFR, RELB, PTPRN, HIF1A, ARRB1, VEGFA, MAPK3, PYGO1, RIPK2, CRTC3, WNT5A, GREM1, LIF, VDR, TCF21, AUTS2, ARNTL2, MAFF, EPAS1, KLF12, MET, SMYD3, SMAD3, TEAD3, NOTCH3, HDAC5, ITGA6, EBF3, HOXB5, SALL1, EBF2, EBF1, KLF2, ENG, PPARA, ACVRL1, PPARG, HEXB, PRRX1, ZEB1, NFKB2, GLI2, PAX8, ALX4, FGF1, FGF2, TNIP1, FOSL1, CYR61, NR2F1, AR, ARHGEF2, HMGA2, HMGA1, PRKD1, INHBA, ADRB2, ZMIZ1, HIPK2, FOXC2, FOXC1, TBX19, CSF3, GLIS3, LUM, PML, NFIX, EGLN1, DCN, NPAS2, POU2F2, PPP3CB, BCL3, ETV6, FOXD1, FZD8, IL6, FOXL2, CEBPB, STAT1, CDH13, HIF3A, APBB1 | 1.85E-04 |
|  | GOTERM_BP_DIRECT | GO:0007165~signal transduction | 113 | DLC1, MOK, MPZL1, PPP2R5A, CRABP2, FST, FGF11, RRAD, ITPKB, PRKG1, CXCL12, GDNF, GRIN2D, STARD8, IL1B, RAPGEF1, EGFR, MAGI3, PLXNB1, PIK3CD, IRS1, PLAUR, THBD, HIF1A, ARRB1, RIPK2, EXT1, NEK6, TRAF1, FGD1, LITAF, CXXC5, GREM1, CD74, IRAK4, VDR, TEK, CDC42EP3, GABRE, EPAS1, MET, ECM1, GAS6, CBLB, MYO10, PENK, CD274, ABL2, PLAU, FGF7, ACVRL1, PPARG, SDC4, KCNIP3, ARHGAP22, WISP2, TNFRSF11B, PTGES, IL4R, CNTNAP1, FGF1, FGF2, AKT3, NR2F1, AR, RSU1, MPP1, NRXN2, PRKCH, TLE1, ARHGAP24, HMGA2, ARHGAP23, PRKD1, ARHGAP31, DOK1, ADM, TNFRSF10D, RIN2, SRGAP1, PLPP5, CXCL1, TNFRSF21, CCK, CXCL5, C3, CXCL8, GNG11, IL7R, STARD13, CCL26, RGMB, LPXN, PPP3CB, SH2B3, CD4, TRIP10, TRHDE, AXL, RCAN1, ANXA5, APPL2, SH3BP5, LEP, CSNK1E, RPS6KA2, GNG10, HBEGF, GDF15, APBB1, IGFBP4, IGFBP5, BCAR3 | 2.79E-04 |
|  | GOTERM_BP_DIRECT | GO:0008360~regulation of cell shape | 27 | DLC1, FGD1, CCL3, SHROOM3, HEXB, GNA12, ATP10A, ITGB2, LPAR1, TPM1, RHOU, CDC42EP2, MSN, WIPF1, CDC42EP3, FN1, ICAM1, FMNL3, IL6, PLXNB1, FBLIM1, MYH9, MYO10, CSNK1E, FYN, VEGFA, ITGA7 | 3.27E-04 |
|  | GOTERM_BP_DIRECT | GO:0043547~positive regulation of GTPase activity | 66 | DLC1, FGF5, A2M, DENND5A, FGF7, GDNF, ARHGAP22, PTGIR, GBF1, GRIN2D, STARD8, AGAP1, DENND5B, FGF1, RAPGEF1, ERRFI1, FGF2, EGFR, ICAM1, F11R, ARHGEF2, RSU1, PLXNB1, ARHGEF6, SIPA1L3, ARHGEF17, ARHGAP24, ARHGAP23, IRS1, ARHGAP31, NDEL1, ARRB1, PDGFRA, RIN2, RAPGEFL1, SRGAP1, SNX18, FGFR1, FGD1, CCL3, RALGPS2, ARFGAP3, DENND2A, STARD13, CCL26, RGS10, PLEKHG3, CDC42EP2, TEK, CAMK2D, TRIP10, RAP1GAP2, CDC42EP3, ARHGEF10L, IQSEC1, TBC1D2, ARHGDIB, TRIO, SFRP1, ITGA6, FYN, RGS3, RGS4, SPTBN2, HBEGF, BCAR3 | 3.66E-04 |
|  | GOTERM_BP_DIRECT | GO:0007229~integrin-  mediated signaling pathway | 22 | ADAM23, BCAR1, ITGA11, ITGA10, ITGB5, ITGA2, ITGB2, ITGA3, ITGB3, MYH9, PRKD1, FBLN1, DAB2, ITGA6, CTGF, ITGA5, SEMA7A, ITGA8, ITGA7, ADAMTS10, ADAM33, ADAM15 | 4.91E-04 |
|  | GOTERM_BP_DIRECT | GO:0050900~leukocyte migration | 24 | B4GALT1, ICAM1, F11R, PODXL, ITGA3, ITGB2, ITGB3, MYH9, SIRPA, CD74, GAS6, MMP1, SLC16A3, THBD, ITGA6, CD44, ITGA5, FYN, TEK, COL1A2, COL1A1, MSN, JAM2, FN1 | 0.001257 |
|  | GOTERM_BP_DIRECT | GO:0010595~positive regulation of endothelial cell migration | 14 | NRP2, WNT5A, BCAR1, EDN1, ITGB3, PRKD1, TEK, CCBE1, VEGFA, FOXC2, ATOH8, THBS1, FGF1, FGF2 | 0.003106 |
|  | GOTERM_BP_DIRECT | GO:0007507~heart development | 29 | NRP2, PPARA, PAM, EDN1, PPARG, OXTR, BICC1, GLI2, TGFB2, CITED2, ZFP36L1, ECE1, FOXF1, TEK, PPP3CB, HEG1, LOX, SH3PXD2B, FOXL1, MICAL2, TGFBR2, ITGA3, TAB2, ADM, XIRP2, RPS6KA2, SALL1, FOXC2, FOXC1 | 0.006588 |
|  | GOTERM_BP_DIRECT | GO:0050731~positive regulation of peptidyl-  tyrosine phosphorylation | 18 | CSF3, ICAM1, ARHGEF2, IL6, CCK, FGF7, ENPP2, CSPG4, ITGB3, CD74, IL11, LIF, CD44, ITGA5, VEGFA, RIPK2, CD4, EHD4 | 0.009281 |
|  | GOTERM_BP_DIRECT | GO:0001501~skeletal system development | 24 | SH3PXD2B, ALPL, FGFR1, HEXB, NPR3, GLI2, EXTL1, GJA5, ANKH, EPHA2, PRELP, TGFB2, VDR, TNFRSF11B, COL1A2, VCAN, FOXC1, COL1A1, PAPSS1, ALX4, EXT1, PAPSS2, IGFBP4, PITX1 | 0.010004 |
|  | GOTERM_BP_DIRECT | GO:0001701~in utero embryonic development | 29 | FGFR1, ACVRL1, EDN1, ARNT2, GNA12, BCL2L1, GLI2, TPM1, GATA6, FOXF1, APBA2, HEG1, SEC24D, FOSL1, APBA1, MAFF, AR, WDTC1, TGFBR2, SMAD3, MYH9, MGAT1, MAN2A1, ZMIZ1, VEGFA, AMOT, FOXC1, KLF2, RCN1 | 0.010096 |
|  | GOTERM_BP_DIRECT | GO:0010628~positive regulation of gene expression | 36 | CCL3, WNT16, LRRC32, CSF1, VIM, PAWR, RIMS2, IL7R, RIMS1, TGFB2, CITED2, VDR, ACTG2, CTGF, IL1B, MSN, FOXD1, FN1, AR, IL6, PIK3CD, SMAD3, TLE1, ITGA3, HMGA2, PTGFR, GAS6, INHBA, FBLN1, HIF1A, PIAS3, RPS6KA2, VEGFA, PRDM1, ENG, NGF | 0.010117 |
|  | GOTERM_BP_DIRECT | GO:0022617~extracellular matrix disassembly | 17 | SH3PXD2B, A2M, HSPG2, DCN, TIMP2, CAPN2, MMP2, MMP1, CD44, HTRA1, LAMC2, LAMC1, FBN2, ENG, ADAMTS5, FN1, ADAM15 | 0.014227 |
|  | GOTERM_BP_DIRECT | GO:0008284~positive regulation of cell proliferation | 53 | FGF5, FGF7, ARNT2, EDN1, GDNF, IL11, TGFB2, S1PR2, AKR1C2, MYOCD, CTGF, CLCF1, HLX, PDGFC, FGF1, FOSL1, FGF2, DPP4, EGFR, AR, IRS1, MARK4, TNS3, ADM, CCND2, HIPK2, VEGFA, PDGFRA, HAS2, LAMC2, CSF3, FGFR1, CCK, CXCL5, CSF1, CD248, BCL2L1, IL34, GREM1, LIF, ZNF703, THBS1, RUNX2, FN1, IL6, TBX2, TGFBR2, PTGFR, LEP, SFRP1, HBEGF, ADRA1D, F2R | 0.014751 |
|  | GOTERM_BP_DIRECT | GO:0048661~positive regulation of smooth muscle cell proliferation | 15 | EGFR, IL6, TGFBR2, EDN1, ITGA2, STAT1, NOTCH3, IRAK4, CDH13, AKR1B1, TGM2, CAMK2D, HBEGF, THBS1, FGF2 | 0.014908 |
|  | GOTERM_BP_DIRECT | GO:0043066~negative regulation of apoptotic process | 52 | IER3, NUAK2, ARNT2, BNIP3, GLI2, GDNF, CITED2, DAB2, CD44, GATA6, FAM129B, CYR61, EGFR, SOCS3, HMGA2, PLAUR, FMN2, TNFRSF10D, CCND2, VEGFA, SERPINB2, RIPK2, NGF, TNFRSF6B, WNT5A, LIMS2, FHL2, BCL2L1, GREM1, CD74, TEK, DAD1, TGM2, BCL3, HSPA5, THBS1, ANGPTL4, IL6, SMAD3, ANXA5, PTGFR, GAS6, PPIF, LEP, NOTCH2, CDKN1A, PLK3, SFRP1, DUSP1, PLK2, CD59, APBB2 | 0.015833 |
|  | GOTERM_BP_DIRECT | GO:0001649~osteoblast differentiation | 20 | ALPL, CCL3, RRBP1, ITGA11, FHL2, GLI2, SNAI1, EPHA2, PENK, SFRP1, RRAS2, SEMA7A, CREB3L1, COL6A1, VCAN, COL1A1, IGFBP3, RUNX2, IGFBP5, CYR61 | 0.018576 |
|  | GOTERM_BP_DIRECT | GO:0071230~cellular response to amino acid stimulus | 13 | EGFR, CEBPB, COL4A1, CPEB1, ZEB1, BCL2L1, CAPN2, MMP2, SH3BP4, COL1A2, COL6A1, PDGFC, COL1A1 | 0.024658 |
|  | GOTERM_BP_DIRECT | GO:0048844~artery morphogenesis | 9 | NOTCH3, ZMIZ1, VEGFA, PRRX1, FOXC2, FOXC1, PRDM1, ENG, GJA5 | 0.027905 |
|  | GOTERM_BP_DIRECT | GO:0035987~endodermal cell differentiation | 10 | INHBA, COL4A2, ITGA5, ITGA7, ITGB5, COL6A1, ITGB2, HMGA2, MMP2, FN1 | 0.028618 |
|  | GOTERM_BP_DIRECT | GO:0001570~vasculogenesis | 14 | TGFBR2, EPHA2, GJC1, CITED2, ZFP36L1, MYOCD, ADM, ZMIZ1, FOXF1, VEGFA, HEG1, AMOT, HAS2, ENG | 0.033189 |
|  | GOTERM_MF_DIRECT | GO:0001968~fibronectin binding | 13 | CCDC80, FSTL3, ITGA3, ITGB3, SDC4, FBLN1, CTGF, VEGFA, THBS1, IGFBP3, SSC5D, PLEKHA2, IGFBP5 | 1.20E-05 |
|  | GOTERM_MF_DIRECT | GO:0050840~extracellular matrix binding | 12 | BGN, CD248, TGFBI, VEGFA, OLFML2A, DCN, ITGB3, THBS1, SSC5D, ADGRG1, ADAMTS5, CYR61 | 1.57E-04 |
|  | GOTERM_MF_DIRECT | GO:0005178~integrin binding | 23 | EGFR, ICAM1, ADAM23, ACTN1, ITGA2, MFGE8, ITGA3, EDIL3, TIMP2, S1PR2, FBLN1, WISP2, ITGA6, CTGF, ITGA5, SEMA7A, TGFBI, THBS1, FGF1, ADAMTS5, FN1, CYR61, ADAM15 | 2.62E-04 |
|  | GOTERM_MF_DIRECT | GO:0005515~protein binding | 596 | DLC1, LDHA, SLC9A7, A2M, LTBP1, MASP1, LTBP2, PPP2R5A, RUSC2, FSTL3, TLDC1, AMOTL1, AMOTL2, KIFC3, CITED2, MAP3K5, FLI1, CTGF, GRIN2D, SERPINE1, PTPRJ, LRRC3, MAGI3, MAGI1, TTC7B, PIK3CD, ZHX3, SERPING1, PTPRU, PTPRN, MYH9, ERGIC1, MARK4, PLAUR, ADAMTS7, GLUL, TAGLN, F3, MAPK3, FOXG1, FCHSD2, ADAMTS5, TNFRSF6B, DCBLD2, IL1R1, DBNDD2, NFKBIE, BCL2L1, FAM212A, CTIF, HSPA1L, PPP1R3C, CDC42EP2, UBASH3B, GORASP2, AHNAK2, IQSEC1, SGIP1, KLF12, IL1RN, TGFBR2, GAS1, EMILIN2, GAS6, KRTAP4-12, RGS3, SYTL4, MAP4, SMURF2, MAP6, LIMA1, FGF7, NUAK2, FAM3C, ATP10A, BNIP3, AP3S1, CDR2L, KRT33B, IL4R, HLX, TICAM2, FGF1, ERRFI1, FGF2, DPP4, SH3PXD2B, AR, ARHGEF2, ACO1, ARHGEF6, FBLIM1, TLE1, FLNC, CARD10, DOK1, INHBA, FAM167A, FAM114A1, CLIC4, CCND2, CYBRD1, SORT1, FOXC2, FOXC1, VCAN, ARL4D, ARL4C, SRGAP1, ALPL, ALDH18A1, TBC1D9, C3, REPS1, FHL1, CSF1, PML, HK1, FHL2, IL7R, AZIN2, PPP3CB, COL6A2, WIPF1, PTX3, TBC1D1, TRIP10, TBC1D2, PPP1R18, CPT1B, IL6, SLC8A1, NTNG1, AXL, NID2, CAPN2, APPL2, DUSP5, PLEKHF1, VAT1L, CDH15, LRP1, DUSP1, RPS6KA2, CSNK1E, GNG10, MT2A, SH3RF3, ZBTB4, DYM, SH3RF2, AMOT, MEGF6, PHLDB2, MPZL1, GNA12, RRAD, KANK1, IL17RA, IL11, TGFB2, DAB2, ELOVL5, SLC2A1, TGFBI, SMIM3, RRAS, ASPH, SPON2, EGFR, CHAC1, RELB, STIM1, ACTN1, TMSB10, IRS1, PKIA, TACC1, RBPMS, HIF1A, THBD, HSPB6, ARRB1, HSPB8, HSPB7, COL1A2, TESK2, COL1A1, NFE2L3, TNFAIP3, SSC5D, NGF, TRAF1, PAM, ARFGAP3, CCL3, LITAF, ADORA2A, TNFRSF12A, STK10, MRAS, BBS9, IL32, CXXC5, TIMP2, TIMP3, VDR, P2RY6, ECE1, NUMB, TEK, UPK2, ARHGDIB, ANGPTL4, EPAS1, ADAM23, EFEMP2, EFEMP1, MET, NTN4, EFS, TEAD3, CYB561, ABCB4, HDAC5, MYO10, CDKN1A, NREP, PLK3, PLK2, SLC7A1, EBF1, PNRC1, PTTG1IP, FBXO32, ABL2, ADRA1D, LRRC8E, IER3, ATL1, LGALS9C, PINK1, GABBR2, PAWR, SDC4, RHOU, MBP, SH2D4A, FRMD6, TRIM8, CLCF1, PAX8, SNTB1, LOXL4, RHOC, LOXL3, LOXL2, FOSL1, SEC24D, CABLES1, AHNAK, AKR1C1, VASN, STXBP1, HMGA2, HMGA1, RFWD2, ADRB2, SDC1, RRAS2, CLDN1, FHOD3, JAM2, TM4SF4, SNX18, VIM, BTN2A2, EGLN1, CDH2, DCN, GRAMD3, NAALADL2, HIC1, SEC22B, SH2B3, ADAMTS10, CD4, HSPA5, FBN2, ADAMTS12, UBTD1, EHD4, WDTC1, COL4A2, CEBPB, ADARB1, COL4A1, C1ORF21, GYG1, PROSER2, ANXA5, COTL1, TAB2, AVPI1, AJUBA, SNX33, RCN3, HBE1, GDF15, THEMIS2, RCN1, FEZ1, PRR16, SEC31A, EDN1, FAM20C, FST, GDF5, PDLIM2, LPAR1, PRKG1, ZNRF1, S1PR2, ATP2B4, CD44, GBF1, HOMER3, IFT20, SEMA7A, WNK4, RAB29, NT5C2, CCBE1, TRAK1, CREB3L1, BCL7A, RAPGEF1, PITX1, RECK, MATN2, F11R, EFNB1, EMX2, PRDM8, KRT19, TACSTD2, PIAS3, KRT15, KRT14, PGM1, VEGFA, PDGFRA, PDE4DIP, TGFB1I1, PRDM1, NEK6, MME, BDKRB1, BDKRB2, RIMS2, RIMS1, CD74, CALU, LIF, SLC11A2, TRAM2, DRAP1, CDA, RAP1GAP2, PLAT, TRPC4, PTPN14, NOTCH2NL, PTPN13, SMAD3, TRIO, MYOZ3, CSRP2, ECM1, EPHA2, NOTCH3, NOTCH2, LAMA1, CBLB, SRPX2, SFRP1, CD59, KLF2, ENG, PLAU, PPARA, JPH2, ACVRL1, BCAR1, PPARG, ARSI, CDCP1, NFKB2, ZEB1, GLI2, KCNIP3, CEP250, ZFYVE9, HHIP, NR2F1, ICAM1, LY96, PSG1, RAB11FIP5, CDR1, CDR2, DACT1, ADM, PSG5, SUSD6, GADD45B, ADAM15, RAB3B, SLC39A13, CXCL8, LARP1B, FKBP1A, SEC14L1, TPCN1, STARD13, ZFP36L1, LPXN, MORC4, SPEG, FAT1, APBA2, BCL3, BCL6, ETV6, THBS1, FYCO1, MGARP, APBA1, FZD8, SPSB1, TBX2, TECPR2, HSPG2, FZD2, STAT1, SNAI1, MSRB3, BLVRA, SLC16A3, RAB32, DKK1, MAPK13, FYN, GFPT2, APBB2, DRAM1, APBB1, BCAR3, CAST, F2RL2, WFS1, CCDC85B, CRABP2, ARNT2, ITPKB, ANKRD1, UBQLN2, FOXO3, GDNF, MMP2, PTGIS, DYSF, MYOCD, GATA6, PDGFC, LOX, KRT86, SATB1, KRTAP2-3, SOCS3, PLXNB1, SOCS5, FIBCD1, MOXD1, SP140, TNS3, NDEL1, TNS1, PYGO1, DSP, AKAP6, RIPK2, CLIP3, CLIP4, MVP, PRPS1, WNT5A, TSHZ3, FGFR1, SSH1, PEAK1, ITGB5, ITGB2, ITGB3, GREM1, FTH1, IRAK4, ERO1A, CDYL2, ARL6IP5, RUNX2, SRGN, FN1, NFE2, SESTD1, PODXL, ITGA2, LCE2A, ITGA3, PPIF, ITGA6, ITGA5, SALL1, HOXB5, CD274, ITGA7, RFLNA, HABP4, MYLK, PLEKHA2, UBE2E2, TLN2, MLPH, MRVI1, PIFO, ARHGAP22, ANG, ATP8B1, CNTNAP1, MSN, CDK15, TNIP1, AKT3, KIRREL3, IRAK2, C11ORF68, NRIP3, RSU1, MPP1, LDB3, ARHGAP24, PALLD, NLRP1, PRKD1, IFNAR2, CD82, HIPK2, CEMIP, HIPK4, CTSB, TNFRSF21, ZC4H2, LUM, NFIX, C1S, TPM1, TRIB1, NPAS2, TNFRSF1B, ZNF703, ERCC6, SYN1, KRT7, ENO2, CAMK2D, TGM2, SCG5, CNN1, MYOF, SYNPO, TXNIP, FOXL2, MAP1A, NFASC, FRMPD4, PRR5L, PPP1R13L, CCDC68, SH3BP5, SH3BP4, KCNN4, MYPN, IGFBP3, NTM, F2R, IGFBP5 | 5.56E-04 |
|  | GOTERM_MF_DIRECT | GO:0008083~growth factor activity | 28 | CXCL1, CSF3, FGF5, FGF7, CSF1, GDF5, FGF11, IL34, CXCL12, GDNF, IL11, TGFB2, LIF, BDNF, CTGF, CLCF1, PDGFC, FGF1, FGF2, IL6, EFEMP1, LEP, INHBA, DKK1, VEGFA, HBEGF, GDF15, NGF | 0.001466 |
|  | GOTERM_MF_DIRECT | GO:0005201~extracellular matrix structural constituent | 17 | COL4A2, PXDN, COL4A1, LUM, EFEMP2, PRELP, LAMA1, FBLN1, BGN, COL1A2, VCAN, CD4, COL1A1, LAMC1, FBN2, MFAP5, COL8A2 | 0.002011 |
|  | GOTERM_MF_DIRECT | GO:0005518~collagen binding | 16 | LUM, ITGA11, ITGA2, ITGA10, SMAD3, ITGA3, DCN, NID2, ADGRG1, ABI3BP, CD44, CCBE1, TGFBI, CTSB, SRGN, FN1 | 0.002307 |
|  | GOTERM_MF_DIRECT | GO:0008201~heparin binding | 25 | NRP2, FGFR1, FMOD, FGF7, LTBP2, CCDC80, GREM2, ADGRG1, ABI3BP, PRELP, WISP2, SFRP1, SAA1, CTGF, ANG, RSPO3, VEGFA, HBEGF, LAMC2, THBS1, FGF1, FGF2, ADAMTS5, CYR61, FN1 | 0.036691 |
|  | GOTERM_CC_DIRECT | GO:0005925~focal adhesion | 81 | DLC1, LIMA1, MPZL1, TLN2, BCAR1, GNA12, CSPG4, PDLIM2, PDLIM1, SDC4, RHOU, ARHGAP22, DAB2, CD44, ZNF185, STARD8, SNTB1, RRAS, MSN, DPP4, AHNAK, EGFR, ICAM1, RSU1, ARHGEF2, ACTN1, FBLIM1, ARHGAP24, NEXN, MYH9, FLNC, PALLD, HMGA1, PLAUR, RFWD2, ARHGAP31, TNS3, SDC1, TNS1, RRAS2, MAPK3, TGFB1I1, LIMS2, FHL1, VIM, PEAK1, ITGA11, FHL2, MME, ITGB5, CDH2, ITGB3, NHS, LPXN, FAT1, TEK, NUMB, TGM2, CNN1, HSPA5, NFASC, HSPG2, ITGA2, ITGA3, FZD2, CSRP2, MCAM, ANXA5, CAPN2, EPHA2, AJUBA, CDH13, LRP1, ITGA6, LAYN, ITGA5, CD59, ITGA8, PHLDB2, ENG, PLAU | 3.68E-20 |
|  | GOTERM_CC_DIRECT | GO:0031012~extracellular matrix | 60 | PXDN, LTBP1, LTBP2, EDIL3, MMP2, MMP1, TGFB2, HTRA1, SBSN, SERPINE1, TGFBI, LOXL2, LOXL1, CYR61, MATN2, ZP1, MFGE8, MYH9, PRELP, BGN, F3, COL1A2, DSP, VCAN, LAMC1, TGFB1I1, COL1A1, SSC5D, FMOD, LUM, VIM, FKBP1A, DCN, TIMP2, TIMP3, ABI3BP, COL6A3, TGM2, COL6A2, ADAMTS10, COL6A1, FBN2, ADAMTS12, HSPA5, THBS1, COL8A2, FN1, PLAT, COL4A2, COL4A1, CKAP4, EFEMP1, HSPG2, NID2, EMILIN2, ECM1, LAMA1, FBLN1, SFRP1, MMP23B |  |
|  | GOTERM_CC_DIRECT | GO:0005615~extracellular space | 152 | CTHRC1, LTBP2, MASP1, FAM20C, GDF5, CGB2, EDN1, FSTL3, MMP2, CXCL12, IL11, TGFB2, ACTG2, PTGIS, PAPPA, HTRA1, CTGF, SEMA7A, TGFBI, CCBE1, SERPINE1, IL1B, PDGFC, LOX, SPON2, KRT86, EGFR, STC2, SOGA3, SIPA1L3, ACTN1, SERPING1, MFGE8, PRELP, THBD, TACSTD2, F3, VEGFA, COL1A2, STC1, LAMC2, COL1A1, LAMC1, SSC5D, ADAMTS5, WNT5A, TNFRSF6B, XDH, PAM, CCL3, WNT5B, ENPP2, IL32, TIMP2, GREM1, IL34, GREM2, TIMP3, ABI3BP, IRAK4, LIF, HLA-DRB5, SRGN, ANGPTL4, FN1, B4GALT1, PLAT, PODXL, EFEMP1, IL1RN, CHI3L2, PPFIBP2, KRT34, MCAM, ECM1, ACPP, GAS6, LAMA1, SRPX2, SFRP1, CD59, SPTBN2, ENG, PLAU, FGF5, PXDN, HMSD, HEXB, PRKAG2, ANPEP, KRT33B, WISP2, TNFRSF11B, ERFE, SAA1, ANG, IL4R, SEMA3F, LOXL4, LOXL3, MSN, LOXL2, FGF1, LOXL1, FGF2, VASN, ICAM1, LY96, IFNAR2, MAN2A1, ADM, SEMA4F, SERPINB7, SERPINB2, VCAN, CTSB, ALPL, CSF3, CXCL1, FMOD, WNT16, CCK, CXCL5, C3, CSF1, LUM, CXCL8, DCN, CCL26, C1QTNF5, RNASET2, REN, COL6A3, COL6A2, ENO2, PTX3, THBS1, IL6, HSPG2, AXL, WNT2B, LEP, CDH13, DKK3, FBLN1, DKK1, AKR1B1, HBEGF, METRNL, GDF15, IGFBP3, IGFBP4 |  |
|  | GOTERM_CC_DIRECT | GO:0005578~proteinaceous extracellular matrix | 54 | CTHRC1, PXDN, LTBP1, LTBP2, MMP2, MMP1, TNFRSF11B, WISP2, CTGF, CCBE1, TGFBI, LOX, SPON2, FGF1, CYR61, MATN2, ZP1, OLFML2A, PRELP, ADAMTS7, ADAMTS6, BGN, VEGFA, COL1A2, VCAN, ADAMTS2, ADAMTS5, ALPL, WNT5A, FMOD, WNT16, WNT5B, ADAMTSL1, MAMDC2, LUM, CD248, TIMP2, TIMP3, COL6A3, COL6A2, ADAMTS10, FBN2, ADAMTS12, COL8A2, FN1, ANGPTL4, EFEMP1, EMILIN2, ECM1, WNT2B, LAMA1, FBLN1, SFRP1, MMP23B |  |
|  | GOTERM_CC_DIRECT | GO:0005576~extracellular region | 170 | F2RL2, LYPD1, A2M, LTBP1, MASP1, FST, CGB2, EDN1, GDF5, FSTL3, CSPG4, FGF11, MMP2, GDNF, CXCL12, MMP1, IL17RA, IL11, TGFB2, BDNF, PAPPA, HTRA1, CTGF, SERPINE1, OSCAR, TGFBI, IL1B, PDGFC, LOX, C2CD2, STC2, PLXNB1, ACTN1, SERPING1, MFGE8, PRELP, VEGFA, COL1A2, LAMC2, COL1A1, LAMC1, ADAMTS2, MFAP5, ADAMTS5, NGF, WNT5A, TNFRSF6B, FGFR1, IL1R1, CCL3, WNT5B, PAMR1, IL34, TIMP2, GREM2, FAM19A3, TIMP3, CALU, LIF, C1ORF54, FOLR3, TEK, CDA, HEG1, LFNG, SRGN, FN1, ANGPTL4, PLAT, ADAM23, EFEMP2, MET, EFEMP1, NTN4, NOTCH2NL, EMILIN2, ECM1, GAS6, NOTCH3, NOTCH2, LAMA1, SFRP1, PENK, HEBP1, HABP4, PLAU, NRP2, FGF5, FGF7, MTRNR2L12, FAM3C, ARSI, ARSJ, IL4I1, CDCP1, TNFRSF11B, CLCF1, ERFE, SAA1, ANG, RSPO3, LOXL3, HHIP, FGF1, LOXL1, FGF2, KIRREL3, CYR61, ZP1, PSG2, PSG1, INHBA, IFNAR2, PSG9, BGN, ADM, PSG6, PSG5, SERPINB2, CEMIP, VCAN, CTSB, CSF3, CXCL1, FMOD, WNT16, CCK, CXCL5, C3, LUM, CXCL8, C1S, DCN, IL7R, TNFRSF1B, PLIN2, GLIPR1, RNASET2, REN, COL6A3, COL6A2, COL6A1, SCG5, FBN2, PTX3, FIBIN, THBS1, COL8A2, CRIM1, IL6, COL4A2, COL4A1, HSPG2, LY6K, NID2, PTGFR, WNT2B, LEP, DKK3, FBLN1, DKK1, FAM180A, C5ORF38, HBEGF, MEGF6, GDF15, IGFBP3, IGFBP4, F2R, IGFBP5 |  |
|  | GOTERM_CC_DIRECT | GO:0009986~cell surface | 72 | MICB, MPZL1, ACVRL1, CLSTN2, TRPV2, CSPG4, LPAR1, SDC4, SLC1A4, SRPX, SLC1A3, CD44, CCR10, PDGFC, HHIP, DPP4, PTPRJ, VASN, EGFR, ICAM1, CLMP, HLA-E, ADAMTS7, SDC1, TNS1, BGN, THBD, CLIC4, F3, VEGFA, SORT1, ADAM15, WNT5A, DCBLD2, PAM, IL1R1, WNT5B, TNFRSF12A, ITGB5, ITGB2, ITGB3, GREM1, TIMP2, CD74, SLC11A2, TEK, HSPA5, THBS1, PLAT, TRPC4, MET, AXL, ITGA2, LY6K, ITGA3, MXRA8, KCNK2, EPHA2, NOTCH2, SRPX2, SFRP1, ITGA6, LAYN, ITGA5, CD59, ITGA8, ITGA7, AMOT, HBEGF, ENG, PLAU, F2R |  |
|  | GOTERM_CC_DIRECT | GO:0005886~plasma membrane | 327 | LYPD1, SLC9A7, SLC9A5, GDF5, LPAR1, PRKG1, S1PR2, ATP2B4, CD44, CTGF, HTRA1, HOMER3, GRIN2D, SEMA7A, RAB29, KCNK6, CCR10, OSCAR, SERPINE1, PTPRJ, RECK, F11R, MAGI3, MAGI1, EFNB1, PIK3CD, TTC7B, PTPRU, PTPRN, MYH9, SIRPA, PDCD1LG2, PLAUR, KRT19, F3, PDGFRA, RALGPS2, IL1R1, ENPP2, ITGA11, MME, ITGA10, BDKRB1, CACNB3, BDKRB2, RIMS1, CD74, SLC11A2, RILPL1, CD68, CDC42EP2, HLA-DRB5, AHNAK2, RAP1GAP2, CDC42EP3, SLC28A3, SGIP1, TRPC4, TGFBR2, IL1RN, PTPN13, SMAD3, GAS1, MCAM, KCNK2, EPHA2, ACPP, NOTCH3, NOTCH2, GGT5, CBLB, PENK, SFRP1, RGS3, CD59, RGS4, SYTL4, TCAF2, MAP4, SMURF2, PLAU, NRP2, LIMA1, ACVRL1, JPH2, IL27RA, SLC20A2, TRPV2, BCAR1, ATP10A, CDCP1, KCNJ12, KCNIP3, VEPH1, TNFRSF11B, SLC24A3, TICAM2, FAM129B, DPP4, KCNG1, ICAM1, AR, FMNL3, ZP1, CLMP, GPR176, LY96, RFTN1, FLNC, STX1B, FMN2, CLIC4, SEMA4F, TNFRSF10D, CYBRD1, SERPINB2, SORT1, ARL4D, STEAP2, ARL4C, STEAP1, RASD2, ADAM15, HLA-DQB1, ALPL, SLC39A14, RAB3B, LIMS2, REPS1, C3, CSF1, FHL1, GNG11, CLDN11, IL7R, TPCN1, GJC1, DGKA, FNDC4, LPXN, PLIN2, GLIPR1, FAT1, PPP3CB, APBA2, SLC4A7, DFNA5, SLC8A1, HSPG2, AXL, LY6K, GRIA3, FZD2, NPR3, CAPN2, PTGFR, SLC16A3, CDH13, CDH15, DKK1, LRP1, FYN, DRP2, GNG10, BNC2, HBEGF, APBB1, PHLDB2, F2RL2, MICB, TSPAN5, GNA12, RRAD, UBQLN2, MMP2, IL17RA, KANK1, DAB2, PTGIR, DYSF, SLC2A3, ZNF185, TGFBI, SLC2A1, RRAS, PDGFC, ASPH, EGFR, TMEM204, PLXNB1, CA12, ACTN1, STIM1, MRGPRF, CERCAM, HLA-E, IRS1, ANKRD13A, THBD, ARRB1, DSP, PSCA, CLIP3, WNT5A, FGFR1, PAM, WNT5B, LITAF, SSH1, TNFRSF12A, ADORA2A, MRAS, STK10, PHKA1, PEAK1, ITGB5, ITGB2, ITGB3, IRAK4, P2RY6, ECE1, NUMB, TEK, RHOBTB1, ARL6IP5, B4GALT1, GABRE, MYO1B, ADAM23, PODXL, CKAP4, MET, NTN4, SLC6A15, ITGA2, ITGA3, ANKH, ABCB4, NMT2, MYO10, ITGA6, ITGA5, GPR39, SLC7A1, ITGA8, CD274, ITGA7, ECSCR, SLC13A3, ADRA1D, PLEKHA2, LRRC8E, OPCML, TLN2, GABBR2, PAWR, SDC4, RHOU, MBP, SLC1A4, CRYL1, FRMD6, SLC1A3, ITPRIP, ATP8B1, MGLL, RHOC, ABHD12, MSN, AHNAK, AKT3, ANO10, KIRREL3, VASN, IRAK2, PTGER2, NRXN2, STXBP1, PRKCH, PALLD, SIGLEC15, LYPD6B, PRKD1, IFNAR2, SDC1, ADRB2, CA9, STXBP6, CD82, RRAS2, CEMIP, SLC41A1, PCYOX1, CNTN3, JAM2, PLPP5, TNFRSF21, VIM, OXTR, CDH2, PCDHGC3, GPR4, RGS10, RGMB, TNFRSF1B, REN, ENO2, TGM2, CAMK2D, CD4, HSPA5, MYOF, CRIM1, B4GALNT1, EHD4, NFASC, TAB2, UBL3, CYSTM1, AJUBA, LMBR1L, KCNN4, SLC17A5, TMEM47, NTM, FEZ1, F2R |  |
|  | GOTERM_CC_DIRECT | GO:0070062~extracellular exosome | 229 | A2M, LDHA, LTBP2, FAM20C, RUSC2, PTGS1, PDLIM2, LUZP1, EDIL3, KIFC3, ACTG2, CD44, PLOD1, IFT20, HTRA1, PLOD2, SBSN, RAB29, SERPINE1, OSCAR, DDAH1, PTPRJ, F11R, EFNB1, SERPING1, MYH9, SIRPA, PDCD1LG2, PRELP, PLAUR, MGAT1, KRT19, GLUL, TACSTD2, KRT15, F3, KRT14, MAPK3, PGM1, LAMC1, MGAT5, TPST2, MME, FAM212A, RIMS2, CD74, SERINC2, HLA-DRB5, PLAT, IL1RN, PTPN13, KRT34, ECM1, GAS6, ACPP, GBE1, SFRP1, CD59, HEBP1, MAP4, TPRG1L, PLAU, PXDN, SLC20A2, FAM3C, ANPEP, KRT33B, CEP250, SAA1, FAM129B, DPP4, ICAM1, CLMP, ACO1, RFTN1, BGN, CLIC4, PSG4, CYBRD1, ADAM15, ALPL, RAB3B, C3, CSF1, FKBP1A, CLDN11, RNASET2, FAT1, COL6A3, COL6A2, TBC1D4, COL6A1, ACSL4, THBS1, TRIP10, TRHDE, A4GALT, HSPG2, AXL, MXRA8, NID2, NPR3, CAPN2, APPL2, MID2, BLVRA, CDH13, FBLN1, CDH15, GFPT1, GNG10, AOX1, PRSS23, CRABP2, CSPG4, CXCL12, ST3GAL1, DAB2, DYSF, SLC2A3, SLC2A1, TGFBI, RRAS, IL1B, PDGFC, SPON2, KRT86, ACTN1, MRGPRF, MFGE8, HLA-E, COL1A2, DSP, PSCA, TNFAIP3, MVP, WNT5A, PAM, WNT5B, MRAS, STK10, ITGB5, ITGB2, ITGB3, TIMP2, TIMP3, FTH1, ECE1, ARL6IP5, UPK2, ARHGDIB, FN1, B4GALT1, MYO1B, EFEMP2, CKAP4, PODXL, EFEMP1, BPGM, ITGA3, ENDOD1, REEP2, ADGRG1, ABCB4, CD274, PTTG1IP, SLC13A3, MYLK, OPCML, MLPH, HEXB, SDC4, SLC1A4, CRYL1, WISP2, CLCF1, ANG, SH3D21, LOXL4, RHOC, MSN, AKR1C1, AHNAK, VASN, RSU1, STXBP1, PRKCH, ARHGAP23, VAT1, PADI1, MAN2A1, SDC1, RRAS2, CD82, PCYOX1, CTSB, SNX18, LUM, CD248, VIM, BTN2A2, CDH2, C1S, PCDHGC3, C1QTNF5, KRT7, DAD1, TGM2, ENO2, HSPA5, MYOF, CRIM1, EHD4, GSTA1, FAM26E, COL4A2, NFASC, TMBIM1, GYG1, COTL1, ANXA5, UBL3, CYSTM1, ELFN1, SH3BP4, AKR1B1, METRNL, GDF15, IGFBP3 |  |
|  | GOTERM_CC_DIRECT | GO:0008305~integrin complex | 12 | ITGA6, ITGA5, ITGA8, ITGA7, ITGA11, ITGA10, ITGB5, ITGA2, ITGB2, ITGA3, ITGB3, MYH9 |  |
|  | GOTERM_CC_DIRECT | GO:0005788~endoplasmic reticulum lumen | 31 | WNT5A, ADAMTSL1, WNT5B, ARSI, ARSJ, P3H2, GBF1, P4HA2, ERO1A, RNASET2, COL6A3, COL6A2, COL6A1, GPX8, CD4, PDGFC, HSPA5, THBS1, COL8A2, COL4A2, COL4A1, CERCAM, GAS6, RDH5, PLAUR, ADAMTS7, COL1A2, COL1A1, RCN3, ADAMTS5, RCN1 |  |
|  | GOTERM_CC_DIRECT | GO:0030018~Z disc | 22 | SLC8A1, JPH2, PPP2R5A, SYNC, LDB3, FHL2, ACTN1, FKBP1A, MYOZ3, PALLD, NEXN, FLNC, SYNPO2L, KRT19, ATP2B4, XIRP2, MYPN, FHOD3, PPP3CB, AHNAK2, FBXO32, SYNPO |  |
|  | GOTERM_CC_DIRECT | GO:0005604~basement membrane | 17 | MATN2, COL4A1, EFEMP2, CCDC80, HSPG2, NTN4, NID2, TIMP3, P3H2, LAMA1, FBLN1, ITGA6, TGFBI, LAMC1, LOXL2, COL8A2, LOXL1 |  |

**Table S2 KEGG pathway analysis of differentially expressed genes associated with colorectal cancer**

| expression | Term | Count | genes | FDR |
| --- | --- | --- | --- | --- |
| Up-regulated DEGs | hsa04110:Cell cycle | 20 | E2F1, CDC6, E2F2, TP53, PKMYT1, CDC20, SFN, MCM2, CDC25C, MCM3, MCM4, CDC25A, MCM5, MCM6, CDC45, MCM7, PLK1, ORC1, STAG2, TFDP1 | 0.016025 |
|  | hsa04974:Protein digestion and absorption | 16 | SLC8A2, ATP1B2, COL3A1, COL15A1, SLC7A8, COL2A1, ATP1A2, COL5A2, COL14A1, COL9A3, COL27A1, CPA2, CPB1, COL24A1, COL11A1, COL10A1 | 0.03695 |
| Down-regulated DEGs | hsa05205:Proteoglycans in cancer | 42 | WNT5A, FGFR1, WNT16, WNT5B, MRAS, LUM, ITGB5, DCN, ITGB3, SDC4, MMP2, TIMP3, TGFB2, CD44, CAMK2D, RRAS, MSN, THBS1, FGF2, AKT3, FN1, EGFR, FZD8, MET, PIK3CD, HSPG2, ITGA2, FZD2, FLNC, PLAUR, WNT2B, CDKN1A, SDC1, CBLB, HIF1A, ITGA5, MAPK13, RRAS2, MAPK3, VEGFA, HBEGF, PLAU | 3.24E-08 |
|  | hsa05200:Pathways in cancer | 62 | FGF5, FGF7, GNA12, PPARG, ARNT2, FGF11, LPAR1, NFKB2, GLI2, MMP2, CXCL12, MMP1, TGFB2, PAX8, SLC2A1, HHIP, FGF1, FGF2, AKT3, EGFR, AR, PTGER2, PIK3CD, HIF1A, MAPK3, VEGFA, PDGFRA, LAMC2, LAMC1, TRAF1, WNT5A, FGFR1, WNT16, WNT5B, PML, CXCL8, GNG11, EGLN1, BDKRB1, BCL2L1, BDKRB2, TCF7L1, FN1, FZD8, COL4A2, IL6, COL4A1, EPAS1, MET, TGFBR2, SMAD3, ITGA2, ITGA3, FZD2, STAT1, WNT2B, LAMA1, CDKN1A, CBLB, ITGA6, GNG10, F2R | 1.12E-07 |
|  | hsa04512:ECM-receptor interaction | 26 | ITGA11, ITGB5, ITGA10, ITGB3, SDC4, CD44, COL6A3, COL6A2, COL6A1, THBS1, FN1, COL4A2, COL4A1, HSPG2, ITGA2, ITGA3, LAMA1, SDC1, ITGA6, ITGA5, ITGA8, ITGA7, COL1A2, LAMC2, LAMC1, COL1A1 | 1.63E-07 |
|  | hsa04151:PI3K-Akt signaling pathway | 56 | FGF5, FGF7, PPP2R5A, FGF11, FOXO3, LPAR1, IL4R, CREB3L1, PDGFC, FGF1, FGF2, AKT3, EGFR, PIK3CD, IRS1, IFNAR2, CCND2, MAPK3, VEGFA, COL1A2, PDGFRA, LAMC2, LAMC1, COL1A1, NGF, CSF3, FGFR1, CSF1, ITGA11, ITGA10, ITGB5, GNG11, BCL2L1, ITGB3, IL7R, TEK, COL6A3, COL6A2, COL6A1, THBS1, FN1, COL4A2, IL6, COL4A1, MET, ITGA2, ITGA3, EPHA2, LAMA1, CDKN1A, ITGA6, ITGA5, GNG10, ITGA8, ITGA7, F2R | 3.76E-07 |
|  | hsa04510:Focal adhesion | 39 | TLN2, BCAR1, ITGA11, ITGB5, ITGA10, ITGB3, COL6A3, COL6A2, COL6A1, PDGFC, THBS1, RAPGEF1, AKT3, FN1, EGFR, COL4A2, COL4A1, PIK3CD, MET, ACTN1, ITGA2, ITGA3, CAPN2, FLNC, LAMA1, ITGA6, FYN, ITGA5, CCND2, ITGA8, MAPK3, ITGA7, VEGFA, COL1A2, PDGFRA, LAMC2, COL1A1, LAMC1, MYLK | 4.36E-06 |
|  | hsa04810:Regulation of actin cytoskeleton | 38 | FGFR1, FGF5, FGD1, FGF7, SSH1, MRAS, DIAPH2, BCAR1, GNA12, ITGA11, FGF11, ITGB5, ITGA10, BDKRB1, ITGB2, ITGB3, BDKRB2, RRAS, PDGFC, MSN, FGF1, FGF2, FN1, EGFR, ARHGEF6, PIK3CD, ACTN1, ITGA2, ITGA3, ITGA6, ITGA5, RRAS2, ITGA8, MAPK3, ITGA7, PDGFRA, MYLK, F2R | 2.93E-05 |
|  | hsa04015:Rap1 signaling pathway | 34 | FGFR1, FGF5, FGF7, TLN2, ADORA2A, MRAS, CSF1, BCAR1, FGF11, ITGB2, ITGB3, LPAR1, TEK, RRAS, PDGFC, FGF1, THBS1, RAPGEF1, FGF2, AKT3, EGFR, MAGI3, MAGI1, PIK3CD, MET, SIPA1L3, EPHA2, PRKD1, MAPK13, MAPK3, VEGFA, PDGFRA, F2R, NGF | 0.002355 |
|  | hsa04068:FoxO signaling pathway | 25 | PRKAG2, BNIP3, FOXO3, IL7R, TGFB2, HOMER3, BCL6, AKT3, EGFR, IL6, TGFBR2, PIK3CD, SMAD3, IRS1, CDKN1A, PLK3, PLK2, CSNK1E, CCND2, MAPK13, FOXG1, MAPK3, FBXO32, KLF2, GADD45B | 0.006369 |
|  | hsa04640:Hematopoietic cell lineage | 19 | CSF3, IL6, IL1R1, CSF1, MME, ITGA2, ITGA3, ANPEP, ITGB3, IL7R, IL11, ITGA6, CD44, ITGA5, CD59, IL4R, IL1B, HLA-DRB5, CD4 | 0.009013 |
|  | hsa04380:Osteoclast differentiation | 24 | IL1R1, SOCS3, CSF1, PIK3CD, PPARG, TGFBR2, RELB, FHL2, NFKB2, ITGB3, STAT1, SIRPA, TAB2, TGFB2, IFNAR2, TNFRSF11B, MAPK13, FYN, OSCAR, MAPK3, PPP3CB, IL1B, FOSL1, AKT3 | 0.014163 |
